# Ultrasensitive topological interface modes for refractive index sensing via all-dielectric one-dimensional photonic crystals

**DOI:** 10.1038/s41598-026-47618-z

**Published:** 2026-04-09

**Authors:** Shakeel Ahmed, Muhammad Zeeshan Riaz, Saad Anwar, Maryam Jamil, Juncong Luo, Mi Lin, Zhengbiao Ouyang

**Affiliations:** 1 Key Laboratory of Optoelectronic Devices and Systems of the Ministry of Education of Guangdong Province, College of Physics and Optoelectronic Engineering, Shenzhen University, Shenzhen, 518060 China; 2 Intelligent Optical Measurement Research Institute, Shenzhen University, Shenzhen, 518061 China

**Keywords:** All dielectric PCs, Topological interface modes, Refractive index sensing, Materials science, Optics and photonics, Physics

## Abstract

Topological interface modes (TIMs) are robust, localized states that emerge at the interface between two topologically distinct materials. In this work, the TIMs are studied in a broader frequency spectrum for both TE and TM polarizations using all-dielectric photonic crystal (PC) layers of Si and SiO_2_ in a one-dimensional setting, focusing on their localization, tunability, and robustness. By using the novel phase gradient cancellation (PGC) strategy we combine the two centrosymmetric PCs, P and Q, with the same optical thickness but different topological properties, and achieve highly localized TIMs in the working wavelength window around 1548.6 nm using two distinct odd gaps indicated as Gap-1 and Gap-2. Further extending the proposed design to two schemes for defecting the interface region, i.e., by inserting the defect layer directly at the interface, keeping the two PCs intact, and by removing the terminal layers from the interface of adjacent PCs we achieve highly tunable topological defect modes (TDMs) within the topological operating region. The refractive index (RI) sensing by using these two schemes demonstrate the superior performance under ideal conditions defined by the parameters such as the sensitivity (for the best values) of 397.7 THz/RIU or 3179.56 nm/RIU, the ultra-high-quality factor (Q) of 7.24⤫10^9^, ultra-low detection limit (DL) of 7.6⤫10^− 11^ RIU, and a high figure of merit (FOM) of 1.32 ⤫10^9^ RIU^− 1^. When realistic material absorption and interface roughness (σ = 0–3 nm, Nevot-Croce model) are incorporated, these metrics represent Q ~ 10⁶, DL ~ 10⁻⁷ RIU, and FOM ~ 10⁷ RIU⁻¹, consistent with experimental demonstrations in comparable Si/SiO_2_ structures. This work not only enriches the understanding of TIM phenomena but also paves the way to realize multifunctional and high-performance devices in biomedical applications, optical communications, and integrated photonics.

**Supplementary Information:**

The online version contains supplementary material available at 10.1038/s41598-026-47618-z.

## Introduction

Photonic crystals (PCs) are artificially designed periodic structures with unique properties to control and manipulate light^1^. The existence of the band gap^2^ in PCs enables dynamic tuning to facilitate the design of various photonic components such as sensors^3^, switches^4^, and waveguides^5^. Another key advantage of PCs over existing electronic devices is enhanced computational speed^6^, greater information densities^7^, and lower noise levels^8^. The conventional photonic devices face serious challenges in real-world applications due to defect sensitivity, fabrication imperfections, and losses^9^. For instance, a small variation in the thickness of the crystal layer, the refractive index (RI), or the incident angle (θ_in_) can lead to a remarkable change in the output of the devices^3^.

Topological photonic crystals (TPCs), on the other hand, have emerged as reliable counterparts to conventional PCs^10^. By borrowing the concepts from condensed matter physics, such as topological invariants and topologically protected edge modes, TPCs have transformed the PCs designs successfully, overcoming the fundamental limitations in conventional PCs^11^. TPCs can host defect-immune unidirectional edge modes, enabling loss-resistant light transport. These topologically protected edge modes offer stable photonic wave guiding, making them suitable for protected optical networking, quantum computing, and advanced sensing technologies^11^. While most studies focus on two- (2D) and three-dimensional (3D) topological photonic systems, one-dimensional (1D) TPCs offer advantages, including easy scalability, precise engineering, and compatibility with existing fabrication technologies^3^. The non-trivial band topology in 1D TPCs is characterized by the Zak phase^12^. 1D topological systems circumvent the need for magnetic fields, synthetic dimensions, or cumbersome lattice engineering as required in 2D and 3D platforms, offering a scalable and cost-effective route to realize the robust photonic devices^13^.

Capitalizing on these properties of 1D TPCs, researchers have demonstrated their utility in achieving stable topological interface modes (TIMs) useful in numerous applications such as RI sensing^14^, temperature sensing^15^, and filtering^16^. For instance, a 1D heterostructure composed of Si and SiO_2_ layers has been employed for glucose concentration detection with a sensitivity (S) of 603.75 nm/RIU and quality factor (Q) of 6.33⤫10^4^^17^. A tuberculosis sensor with an S value of 1500 nm/RIU and a Q value of 30659.54 with a detection limit of 2.2⤫10^− 6^RIU has also been proposed^18^. For optical filtering, TIMs have enabled multichannel selective filters with high transmission (T) and narrow linewidths^19^. These advances underscore the potential of 1D TPCs as a multifunctional platform for the next generation photonic devices.

In this work, we present a comprehensive study of all-dielectric centrosymmetric 1D TPCs, focusing on TIMs in two distinct odd gaps, termed as Gap-1 and Gap-2 using the novel phase gradient cancellation (PGC) method. The remarkable stability of TIMs within the topological region under θ_in_ variations from 0–89° highlights their robustness and suitability for practical applications. Furthermore, we extended the design for RI sensing using two defect schemes at the interface: by inserting the defect layer directly at the interface, keeping the two PCs intact (indicated as Scheme-1), and by removing the terminal layers from the interface of adjacent PCs (indicated as Scheme-2). These configurations enabled the tunable topological defect modes (TDMs) defined by the parameters for the best values such as the sensitivity of 397.7 THz/RIU or 3179.56 nm/RIU for 1548.6 nm working wavelength, the high Q of 7.24⤫10^9^, ultra-low detection limits (DL) of 7.6⤫10^− 11^ RIU, and a high figure of merit (FOM) of 1.32 ⤫10^9^ RIU^− 1^. Applying these schemes to RI sensing of brain tumor samples, we demonstrate their potential for biomedical diagnostics with remarkable resolution and stability. Further, we investigate the frequency dependent material absorption and interface roughness using Nevot-Croce model^20,21^, these metrics reduce to Q ~ 10⁶, DL ~ 10⁻⁷ RIU, and FOM ~ 10⁷ RIU⁻¹, consistent with experimental demonstrations in comparable Si/SiO_2_ structures. This work not only enriches the understanding of TIMs phenomena but also lays the foundation of their deployment in biomedical applications, optical communications, and integrated photonics.

## Model and theory

It is well observed that in the multiple photonic bandgaps (PBGs), the TIMs can be excited by band crossing conditions^22^. By stacking the two PCs with different topological charges having the same optical thickness, they can share the common PBG, where TIMs can be excited^23^. To cross the band either at the boundary or at the zone center, it is sufficient to satisfy n_A_d_A_/n_B_d_B_ = m_1_/m_2_, where n_A_ and n_B_ are the RIs of the two materials comprising the layers 1D structure, and m_1_ and m_2_ are the integers. We choose m_1_​ = m_2_​ = 1 (i.e., n_A_​d_A_ ​≈ n_B_​d_B​_) for three specific reasons: (i): Setting quarter-wave Bragg condition n_A_​d_A_ ​≈ n_B_​d_B_ ​≈ c​/4f_o_ ensures strong reflection at the design frequency, creating well-defined PBGs at both the zone center and zone boundary; (ii) The integer ratio guarantees that PCs P and Q possess overlapping common PBGs, enabling the excitation of TIMs at their interface; (iii) Zak phase engineering through specific CPC arrangements P = [B/2 A B/2] and Q = [A/2 B A/2] place inversion centers at high-index (A) and low-index (B) layers, respectively, producing the requisite Zak phase alternation (π, 0, π, 0.) across successive PBGs. This phase alternation ensures that TIMs appear only in odd-numbered gaps (Gap-1, Gap-2, etc.) while remaining absent in even gaps, as dictated by the bulk-boundary correspondence. The specific values d_A_​ = 110 nm and d_B_​ = 270 nm are optimized with unit cell length of both layers is Γ = d_A_ + d_B_ = 380 nm to position the operating wavelength within the telecommunication window while maintaining fabrication compatibility with standard thin-film deposition techniques. The structure is modeled using finite element method (FEM) and validated using transfer matrix method (TMM).

**Fig. 1:**
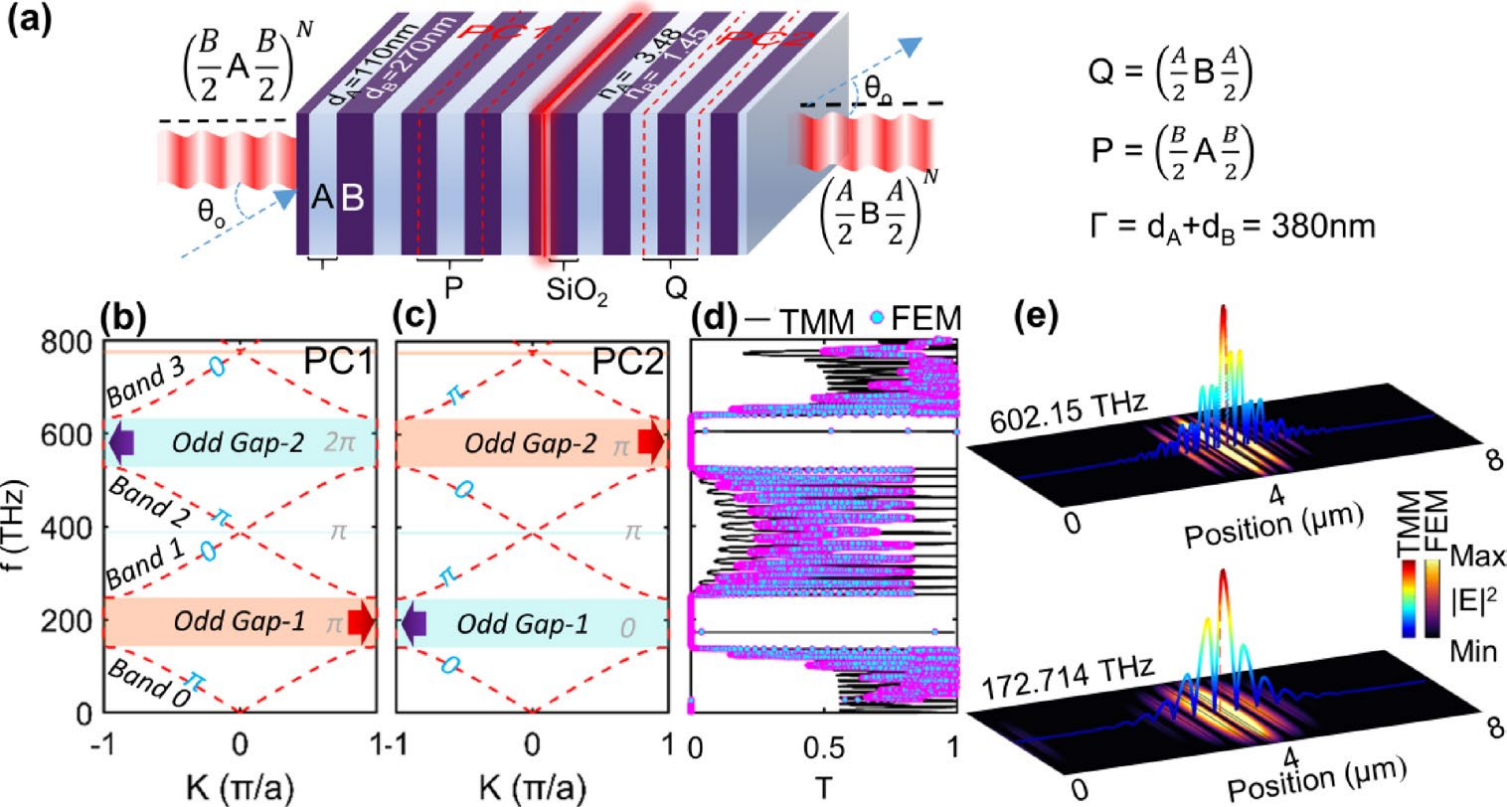
Schematic and properties of the design. **(a)** Proposed model showing the interface between two PCs, P and Q, supporting the TIMs. Light is incident from the left and collected from the right. Photonic band structure of P and Q plotted in **(b)** and **(c)**, respectively. The light-orange strip with red arrows shows the positive topological property, and the cyan strips with purple arrows represent the negative topological property. The Zak phase of each passband is annotated with cyan color in both **(b)** and** (c)**. The numbers annotated with gray in the middle of each PBG represent the sum of Zak phases below each gap. **(d)** T spectra of PQ system with 10 periods each where solid black line is for TMM and markers are for FEM, and **(e)** normalized E field profiles obtained by TMM and FEM for considered TIMs in the first two odd gaps, Gap-1 and Gap-2.

Both layers are assumed to be lossless with negligible dispersion. The band structures of PC_1_ and PC_2_ are presented in Figs. 1(b) and (c) respectively, and the corresponding T spectra of TMM and FEM are presented in Fig. 1(d). Making the light input from the left Port, the TIMs are excited in odd gaps, termed as Gap-1 and Gap-2 in Figs. 1 (b) and (c). The first TIM appears in Gap-1 at 172.714 THz and the second in Gap-2 at 602.15 THz. The corresponding normalized E field profiles for both TIMs are also presented in Fig. 1(e). The photonic band structures for both PCs, P and Q are calculated using the equation^24^

1 $$\:\mathrm{cos}\left(K{\Gamma\:}\right)=\mathrm{cos}\left({k}_{A}{d}_{A}\right)\mathrm{cos}\left({k}_{B}{d}_{B}\right)-\frac{1}{2}\left(\frac{{n}_{A}}{{n}_{B}}+\frac{{n}_{B}}{{n}_{A}}\right)sin\left({k}_{A}{d}_{A}\right)sin\left({k}_{B}{d}_{B}\right),\:\:\:\:\:\:\:\:\:\:\:\:\:\:$$

where K is the Bloch wave vector and Γ is the thickness of the unit cell. It is well known that stacking the two CPCs having the same optical thickness but different topological properties, TIMs can be excited in their common PBGs^22,23^. The spectral position of TIMs is governed by the phase-matching condition at the interface between two CPCs^25^,

2 $$\:\varPhi\:\left(f,\theta\:\right)={\varPhi\:}_{P}\left(f,\theta\:\right)+{\varPhi\:}_{Q}\left(f,\theta\:\right)=2m\pi\:,$$

where, $\:{\varPhi\:}_{L,R}$ are the reflection phases in P and Q stack, *f* is frequency, *θ* is the incident angle and *m* is an integer. This condition defines an implicit curve$\:{f}_{TIM}\left(\theta\:\right)\:\mathrm{i}\mathrm{n}\:\left(f,\:\theta\:\right)\mathrm{p}\mathrm{l}\mathrm{a}\mathrm{n}\mathrm{e}$. Differentiating Equ. (2) with respect to *θ* gives^26^

3 $$\:\frac{{df}_{TIM}}{d\theta\:}=-\frac{{\partial\:}_{\theta\:}{\varPhi\:}_{P}+{\partial\:}_{\theta\:}{\varPhi\:}_{Q}}{{\partial\:}_{f}{\varPhi\:}_{P}+{\partial\:}_{f}{\varPhi\:}_{Q}}\:.$$

Here the term $\:{\partial\:}_{\theta\:}{\phi\:}_{P}+{\partial\:}_{\theta\:}{\phi\:}_{Q}=\partial\:({\phi\:}_{P}+{\phi\:}_{Q})/\partial\:\theta\:$ represents the combined angular derivatives of mirrored phases and $\:{\partial\:}_{f}{\varPhi\:}_{P}+{\partial\:}_{f}{\varPhi\:}_{Q}=\partial\:({\phi\:}_{P}+{\phi\:}_{Q})/\partial\:f$ represents the combined frequency derivative related to group delay from PCs P (Q) and Q (P).

**Fig. 2:**
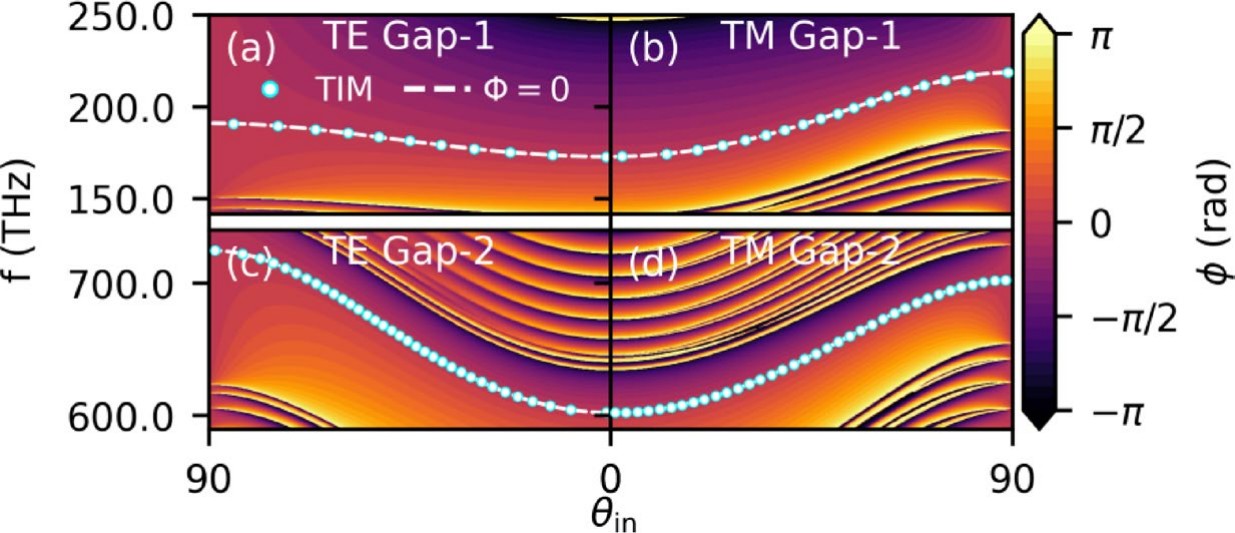
The phase map and TIMs. The combined phase maps corresponding to TE in **(a)**,**(c)** and **(b)**,**(d)** for TM in Gap-1 and Gap-2, respectively, as a function of θ_in_ and frequencies. White lines and markers indicate $\:\varPhi\:=0$ and TIMs, respectively.

Figure 2(a)-(b) represents the phase map for TE and TM for Gap-1 and (c)-(d) for Gap-2 of combined phases from both CPCs, respectively (See Supplementary Note 2 for the detailed individual phase maps of P and Q). The angle-dependence of TIMs can be achieved when$\:\:{\partial\:}_{\theta\:}{\varPhi\:}_{P}\approx\:-{\partial\:}_{\theta\:}{\varPhi\:}_{Q}$ while the frequency derivative remains finite. This mechanism is fundamentally different than the conventional Bragg-gap edge formalism, which provides a mere gap-edge estimate across angular span but does not infer the spectral stability and tunability of TIMs^27^. Additionally, Equ. (3) also emphasizes the materials selection criteria in a particular frequency window as it involves the group delays from both sides of the interface, that are directly influenced by the material response to a particular frequency.

The topological invariant is characterized by the Zak phase in a 1D periodic system. This phase is crucial to understand the optical properties such as polarization and surface impedance of the 1D periodic system and dictates the existence of the TIMs at the interface of two PCs with same optical thickness having the different topological charge. For any 1D system the topological phase is the quantized Zak phase of 0 or π given by^12^

4 $$\:{\theta\:}_{m}^{zak}={\int\:}_{\frac{-\pi\:}{\varGamma\:}}^{\frac{\pi\:}{\varGamma\:}}\left[i{\int\:}_{unitcell}^{}\epsilon\:\left(z\right){u}_{m,k}^{*}\left(z\right){\partial\:}_{k}{u}_{m,k}\left(z\right)dz\right]dK,$$

where ε(z) is the space dependent permittivity, $\:{u}_{m,k}\left(z\right)$ is the Bloch eigen function of the electric field at the mth photonic passband with the Bloch wave vector *K*. Equation (4) represents a geometric phase accumulated by Bloch waves traversing the Brillouin zone in periodic PCs. It is computed by integrating the Berry connection over the crystal momentum space, where the integrand involves the inner product between $\:{u}_{m,k}\left(z\right)$ and its momentum derivative. This topological invariant captures the winding of the electromagnetic field distribution within the unit cell and plays a crucial role in determining the topological properties of PBGs. The presence of the permittivity profile ε(z) in the integral accounts for the material heterogeneity that defines the PC structure. It is observed that in 1D inversion symmetry always possesses two inversion centers^23^. If the Zak phase is 0(π) relative to one inversion center it must refer to π (0) for the other inversion center. The topological sign of each PBG Ф_n_ gives an indication of the presence or absence of the TIMs within the PBG. In the absence of band crossing the Zak phase can be related to Ф_n_^23^_,_ as below

5 $$\:sgn\left[{\varPhi\:}_{n}\right]={(-1)}^{n}\mathrm{e}\mathrm{x}\mathrm{p}\left(i\sum\:_{m=0}^{n-1}{\theta\:}_{m}^{zak}\right).$$

Equation 5 justifies that for all bands and PBGs below the n-th PBG, the Zak phase$\:{\theta\:}_{m}^{zak}$ = π and 0 for m-th band (m ≠ 0). To verify whether there exist TIMs or not at the interface the phases are highlighted in Figs. 1(b)-(c). For the lowest 0th band the Zak phase can be represented as

6 $$\:{\mathrm{e}\mathrm{x}\mathrm{p}(i\theta\:}_{m}^{zak})=sgn\left[1-\frac{{n}_{A}^{2}}{{n}_{B}^{2}}\right].$$

**Fig. 3:**
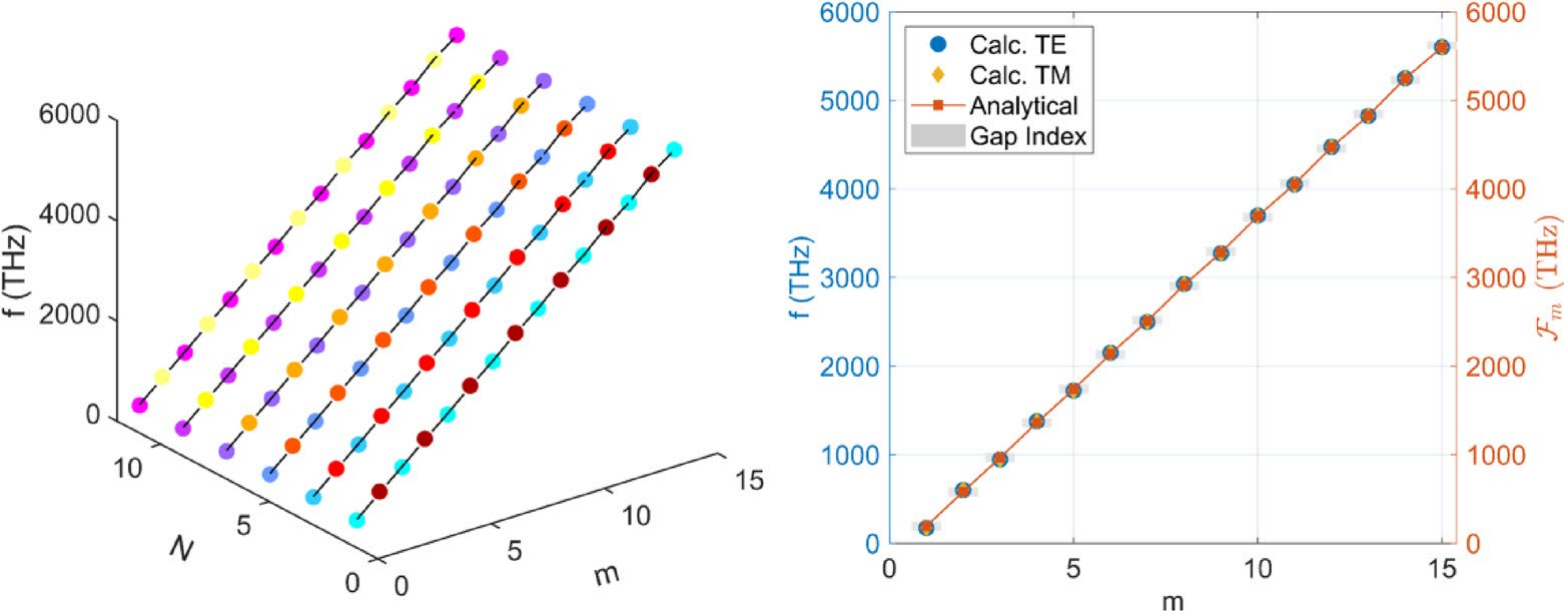
Higher order TIMs. **(a)** Higher order TIM_A_ and TIM_B_ for 2–12 periods. The color gradient represents the number of periods for both TIM_A_ and TIM_B_ where the cool color gradient represents the TIM_A_ and hot color gradient represents TIM_B_ and both colors show saturation towards higher period index N. Both TIMs follow a linear trend with a higher-order frequency spectrum. **(b)** Theoretically calculated and analytically modeled fitting for higher order TIMs according to Equ. (7) for normal incidence.

Equation 6 shows that the Zak phase depends upon the choice of origin and symmetry properties of the band edge mode field^17^, which implies that the same edge mode field leads to a Zak phase of 0 and is π otherwise. The choice of origin plays a crucial role, for instance, the inversion center for a PC with an inversion center at a high RI slab (n_A_> n_B_) possesses the Zak phase of π and 0 for a PC with inversion center at a slab with low refractive index (n_B_> n_A_). This allows for one to design the Zak phases at higher bandgaps to achieve the TIMs at any desired bandgaps^12^. Note that the Zak phase is π and 0 for index contrast (n_A_> n_B_) and (n_B_> n_A_) in Figs. 1(b) and (c) respectively. The Zak phase for higher order bands can be obtained by using the equation,

7 $$\:sin\left(\frac{\omega\:}{c}{n}_{i}{d}_{i}\right)=0,$$

where n_i_ is the RI whose inversion center lies in the i-th (i = A, B) material and ω is the frequency (See Supplementary Note 2 for details). The higher-order patterns of the TIMs and dispersion relation can be found in Supplementary Fig. S1. Figure 3 represents the variation of gap width in the broader frequency regime. Both even and odd gap widths vary steadily as the odd gaps tend to decrease and even gaps tend to increase linearly across the higher-order frequency spectrum.

## Results and discussion

**Fig. 4:**
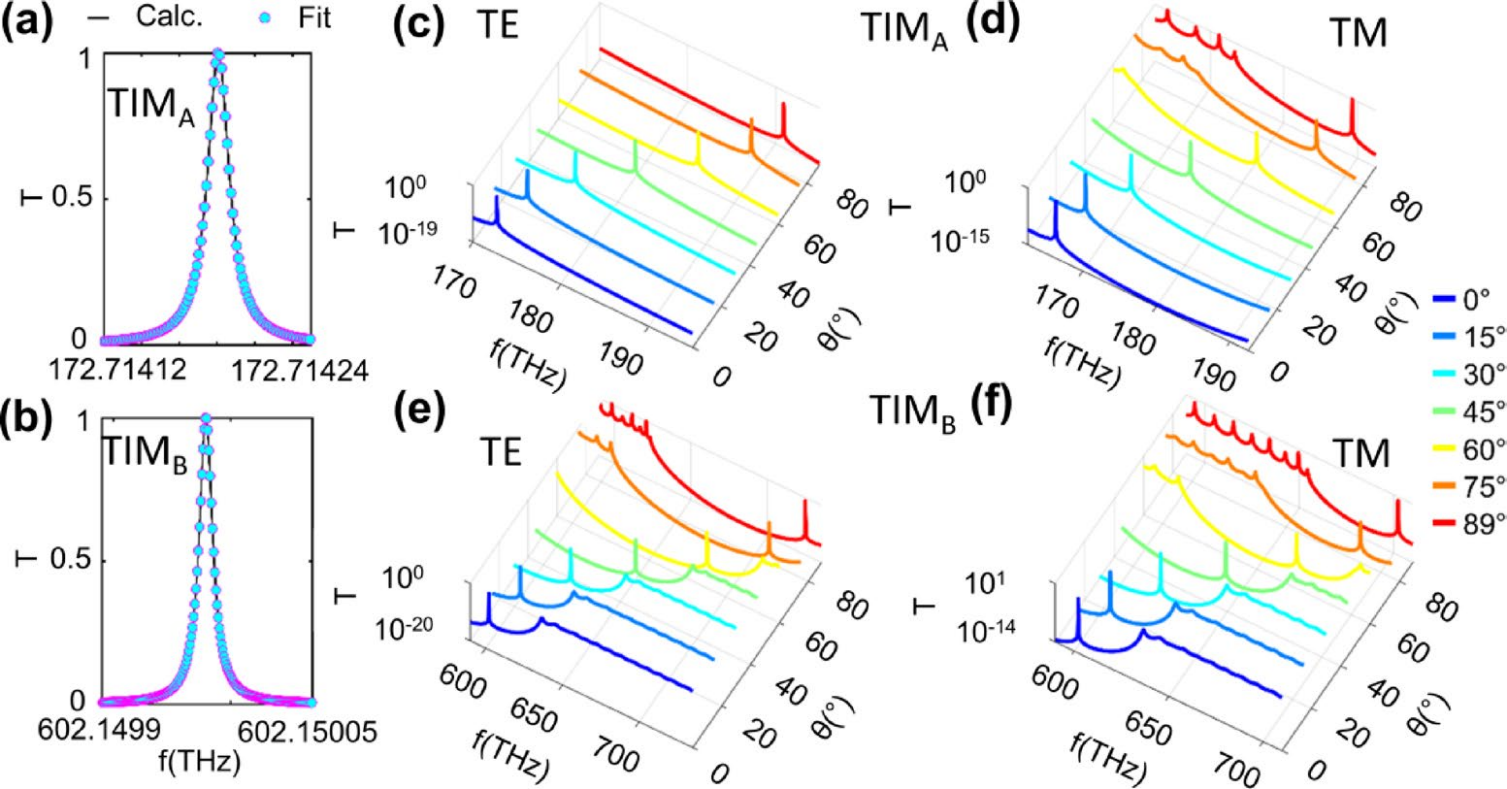
TIMs and their stability. **(a)-(b**) Zoom in profile of TIM_A_ and TIM_B_ (in solid black) respectively showing typical Lorentzian fit in cyan markers according to Equ. (8). **(c)-(d)** T spectra of TE and TM polarizations for TIM_A_ and **(e) –(f)** T spectra of TE and TM for TIM_B_ against the θ_in_ variation from 0°−89°.

Since the optical path lengths of the two centrosymmetric PCs are exactly the same, it becomes interesting to explore the topological properties of such a system. The existence of the TIMs in any PBG can be determined by the sum of the Zak phase below that PBG. In Figs. 1(b) and (c), the sum of Zak phases below each gap is annotated in gray in the middle of the gaps. The Zak phase for the lowest band for P is π, and for Q is 0. The total Zak phase is calculated as $\:{\theta\:}_{Gap2}^{zak}=$ $\:{\theta\:}_{Gap0}^{zak}\left({\uppi\:}\right)+{\theta\:}_{Gap1}^{zak}\left(0\right)$. The sum of Zak phase is different in odd gaps for both P and Q that guarantees the TIMs in these gaps, and is different in even gaps resulting in the absence of TIMs. The detailed information of the Zak phases related to first four gaps is provided in Supplementary Note 2 and Supplementary Table S1. Both TIMs represent typical Lorentzian shape as shown in Figs. 4(a) and (b) respectively. The Lorentz model being used for fitting is given by,

8 $$\:T\left(f\right)=A.\frac{{\left(\frac{\gamma\:}{2}\right)}^{2}}{{(f-{f}_{o})}^{2}+{\left(\frac{\gamma\:}{2}\right)}^{2}},$$

where A is the amplitude of the peak, f_o_ is the resonant frequency, and γ is the full width at half maximum (FWHM). Figures 4(c)-(f) represents the T profiles of TIM_A_ and TIM_B_ for TE and TM modes, respectively. When θ_in_ varied from 0–89°, the TIMs blue shifted within the topological operating regions and became even sharper at the higher slanted angles.

However, the T for TE mode is less than that for the TM mode in both PBGs.

Figure 3(a) explains the existence of alternate TIMs in Gap-1 and Gap-2, where TIM_A_ and TIM_B_ are marked in corresponding colors following the linear trend across the considered frequency spectrum. Interestingly, the TIM_A_ and TIM_B_ appear at the same frequency difference below and above the base frequency $\:{f}_{0}$ i.e., f _TIMA_<$\:{f}_{0}$ in Gap-1 and f _TIMB_>$\:{f}_{0}$ in Gap-2. The TIMs behavior of such a centrosymmetric structure can be explained using the relation:

9 $$\:{\mathcal{F}}_{m}={f}_{0}\left(m\right){\left[1+{\left(-1\right)}^{m+1}\left(A.B\right)exp(-m\frac{1-\alpha\:}{\pi\:})\right]}^{1/2},$$

where $\:{\mathcal{F}}_{m}$ represents the m-th odd TIM, $\:{f}_{0}$ is the base frequency, and A = $\:\frac{{n}_{A}{d}_{A}-{n}_{B}{d}_{B}}{{n}_{A}{d}_{A}+{n}_{B}{d}_{B}}$ is periodic perturbation term that that quantifies to the phase and topological properties within both dielectric materials. α = $\:\frac{{n}_{A}{d}_{A}}{{n}_{B}{d}_{B}}\:$is the optical thickness ratio that explains and formation of TIMs and $\:B=\frac{\alpha\:}{\pi\:}$ normalizes the optical thickness by π linking the phase accumulation within PC structure. The term $\:{\left(-1\right)}^{m+1}$ captures the quasi-periodic behavior of TIMs occurring at alternating frequencies. The model agrees well with the calculated data across the considered frequency spectrum as evident in Fig. 4(b). Based on the properties of the proposed model discussed above, the design can be considered for RI sensing, as discussed in the following sections.

### Defected interface for refractive index sensing

#### Two schemes with defected interface

The aqueous layer as a defect is crucial for sensing applications, which allows one to achieve tunability in certain practical manufacturing and working constraints. Additionally, the stability of dielectric layers in the aqueous environment makes them suitable candidates for bio-sensing applications. To achieve the TDMs in the topological region, the interface is defected via two schemes discussed below. For Scheme-1, we insert an aqueous defect layer right at the interface between P and Q. For Scheme-2, we delete the terminal layers of the adjacent P and Q from the center interface (B/2 layer from P and A/2 layer from Q). The RI of the defect layer is 1.3333 for both schemes. To find the optimized thickness for maximum T in the considered PBGs, the thickness is optimized across the span of 1–1000 nm (see Supplementary Fig. S4).

The T profiles for both schemes are shown in Supplementary Fig. S5. For Scheme-1, the defect thickness is optimized to be 190 nm. When the θ_in_ is varied from 0° to 89°, it is observed that the TDMs for both gaps shift but remain within the topological operating region across the T spectrum, as observed in Supplementary Fig. S5(a)–(h). It is seen that for TDM_A_, the TE and TM modes behave differently. For TE, the T decreases slightly towards higher θ_in_ values, whereas it increases for TM. For TDM_B_, the T behaves similar to that in Gap-1 for TE and TM from 0° to 89°. However, the overall T is slightly lower in Gap-2 than in Gap-1. For Scheme-2, the thickness is optimized to be 380 nm. The TE modes in Gap-1 behave similarly to Scheme-1 but with less T. For TM modes, the T behaves similarly to TE across the θ_in_ scan but with lower values than TE. In Gap-2, the T decreases slightly for higher θ_in_ for TE and increases for TM. The overall T in Gap-2 is more than in Gap-1 for both TE and TM polarizations. The field profiles for TDM_A_ and TDM_B_ are presented in Supplementary Note 5. The field accumulation is towards the Q side in Scheme-1 and the P side in Scheme-2 for both gaps.

#### Refractive index sensing

**Fig. 5:**
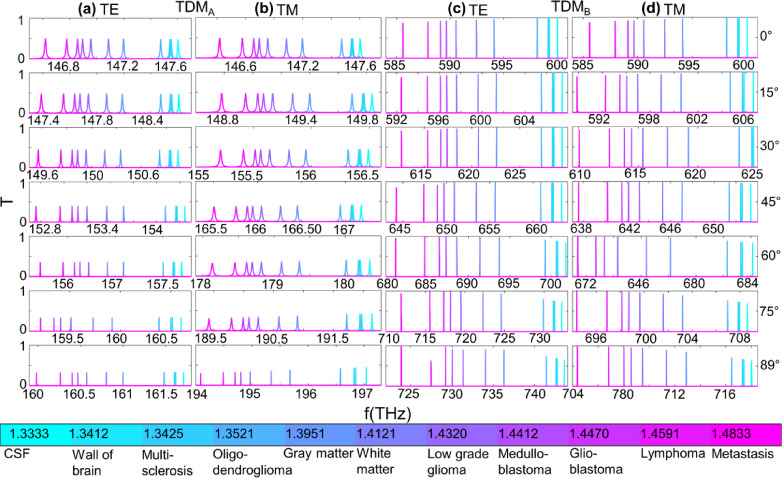
The RI sensing for Scheme-1. **(a)-(b)** The T profiles of TDM_A_ for TE and TM in Gap-1 and **(c)-(d)** of TDM_B_ for TE and TM in Gap-2 respectively.

Using both the schemes discussed above, the RI sensing is carried out for the brain tumor samples as the RI variable in the defect layer. The details of RIs for samples and their categories are provided in the Supplementary Table S3. The brain tumor sample cerebrospinal fluid (CSF) with RI 1.3333 is used as a reference in both schemes. The TDMs are observed to be red-shifted in all observations and for both schemes when the defected interface is loaded with as evident in T profiles in Figs. 5(a)–(d). The TDMs shift but remain within the topological operating region for both TE and TM in both gaps for both schemes.

**Fig. 6:**
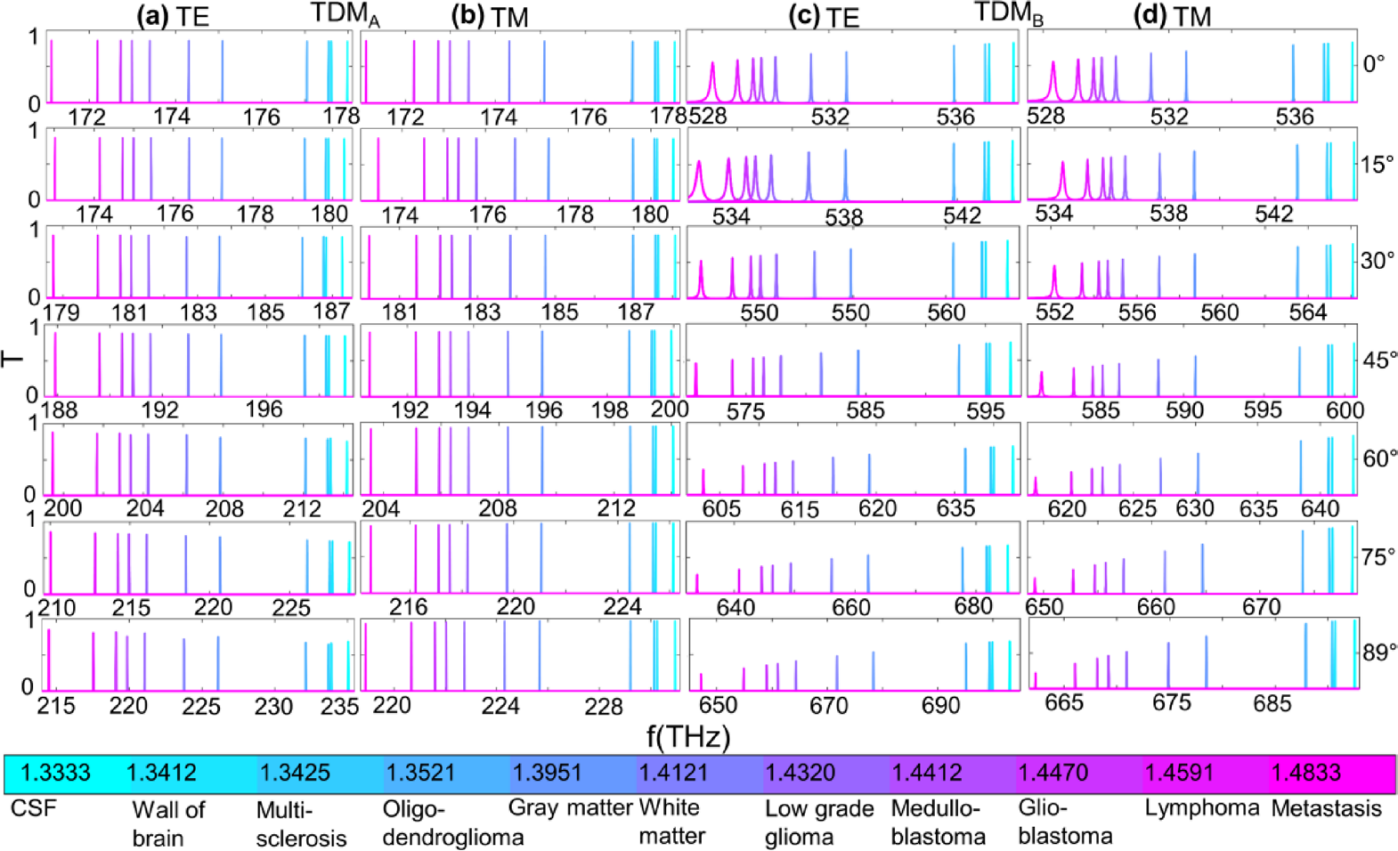
The RI sensing for Scheme-2. **(a)-(b)** The T profiles of TDM_A_ for TE and TM in Gap-1 and **(c)-(d)** of TDM_B_ for TE and TM in Gap-2, respectively.

For Scheme-1, the defect thickness 190 nm is used. The TDM_A_ is red-shifted in the range (146.62 −161.71) THz and (146.62 −197.04) THz for TE and TM, respectively, in Gap-1. In Gap-2, the TDM_B_ red-shifted in the range (585.6 −743.59) THz and (585.6–718) THz for TE and TM, respectively, when the θ_in_ is varied from 0° to 89°. The T for TDM_A_ is 50% for both TE and TM for θ_in_ = 0° and decreases to 40% for θ_in_ = 89°, as can be seen in Figs. 6(a)-(b). The T is lower for the low index region, which is towards CSF, and increases towards the high index region, that is, towards Metastasis for θ_in_ = 0°. For θ_in_ = 89°, this trend alters, that is, the T decreases for the high index region than in the low index region. In Gap-2, the T is high for TDM_B_ both for TE and TM in the low index region for θ_in_ = 0° and gradually increases for θ_in_ = 89° towards the high index region as shown in Figs. 6(c)-(d). Also, the T decreases for the low index region when the θ_in_ is varied from 0° to 89°. In contrast, the Gap-2 shows overall higher T than Gap-1.

For Scheme-2, the defect thickness 380 nm is used. The TDM_A_ is red-shifted as (171.12 −235.05) THz and (171.12 −230.95) THz for TE and TM, respectively, in Gap-1. In Gap-2, the TDM_B_ red shifted as (528.32 −703.02) THz and (528.32 −692.52) THz, when the θ_in_ is varied from 0° to 89°. For TDM_A_ in Gap-1 for TE mode, the T increases with increasing the RIs of the defect layer, as can be seen in Fig. 5(a), and decreases for the low RI region for higher θ_in_ values. For TM, the T increases for all samples for higher θ_in,_ as evident in Fig. 5(b). The decrease in T for the high RI region is rapid as compared to the low RI region for θ_in_ = 89°.

For TDM_B_ in Gap-2, for TM mode, the T decreases with increasing RIs, similar to TE; however, it increases for the low RI region for higher θ_in_ values, as shown in Fig. 5(d). In general, the T is maximum for TM than TE in both Gap-1 and Gap-2, and increases for higher θ_in_ angles in the low RI region.

#### Sensor performance

The sensing parameters such as Q, S, DL, and FOM are crucial to investigate and to understand the sensor performance. These parameters can be described using the Eqs^18^.,

10 $$\:S=\frac{{\varDelta\:f}_{r}}{\varDelta\:n},$$

11 $$\:Q=\frac{{f}_{r}}{FWHM},\:$$

12 $$\:DL=\frac{{f}_{r}}{10\times\:S\times\:Q},\:$$

13 $$\:FOM=\frac{S}{FWHM}.\:$$

In the above equations, Δf_r_ is the change in resonant position of peaks, Δn is the change in RI, and FWHM is the full width at half maximum.

**Fig. 7:**
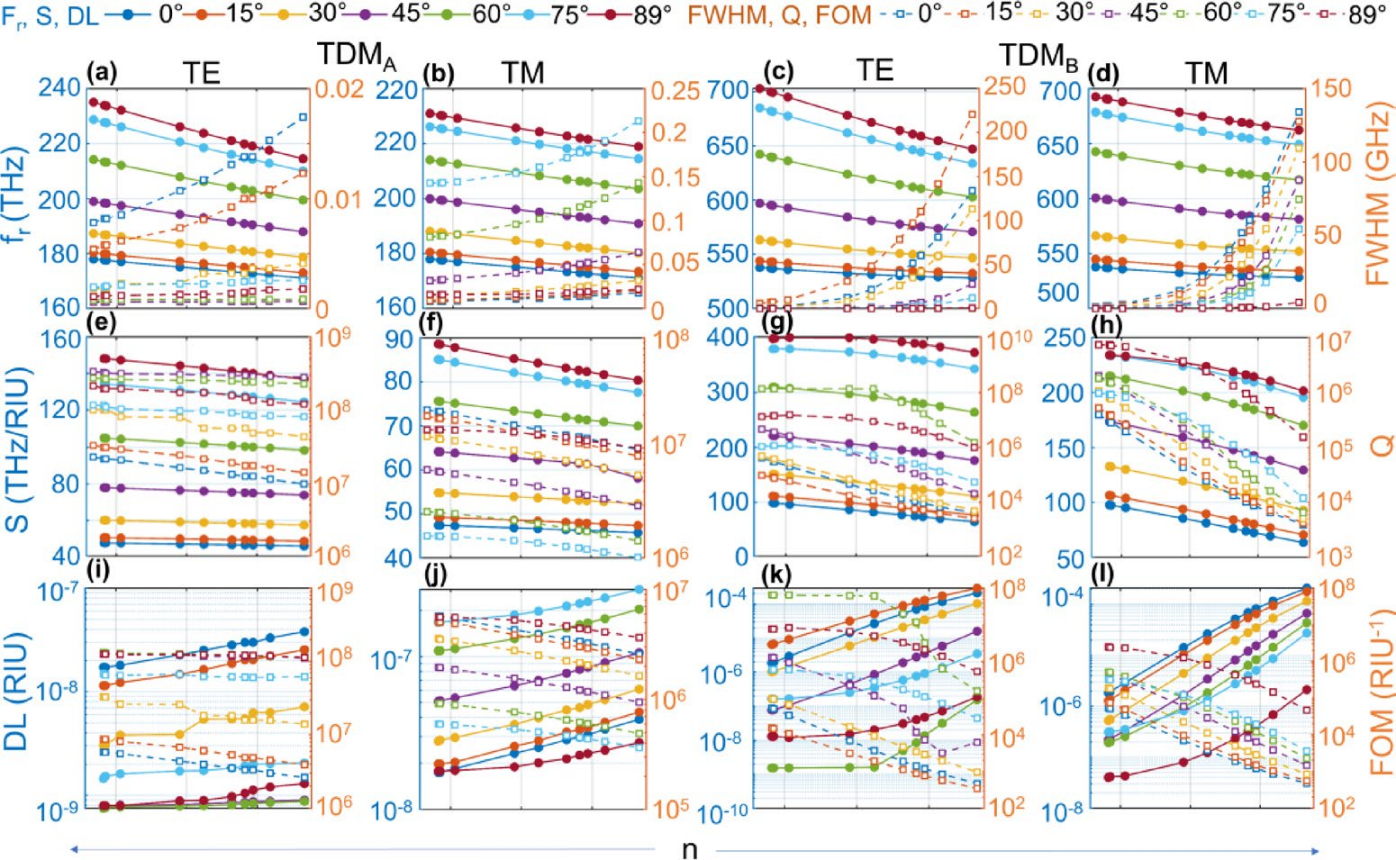
Sensor performance for TDM_A_ and TDM_B_ defected interface in Scheme-2. Variation of sensing parameters for loaded defect, with samples having RIs the same as those in Fig. 8. **(a)–(d)** Resonant frequency vs. FWHM, **(e)–(h)** S vs. Q and **(i)–(l)** DL vs. FOM for TDM_A_ and TDM_B_, respectively. All the solid lines belong to the left vertical axis and all the dashed lines belong to the right vertical axis in **(a)-(l)**.

**Fig. 8:**
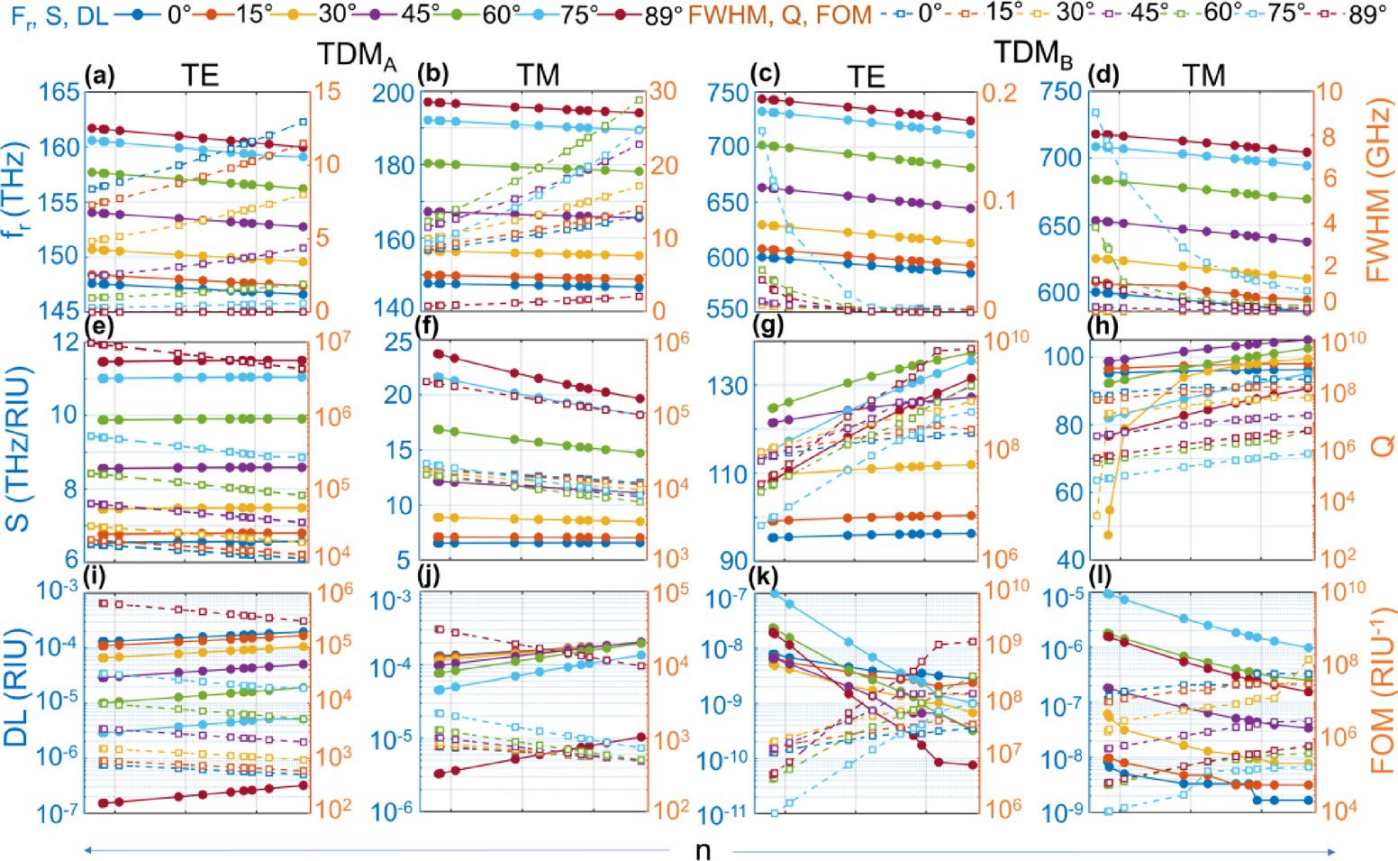
Sensor performance for TDM_A_ and TDM_B_ defected interface in Scheme-1. Variation of sensing parameters for loaded defect, with sample refractive indices from 1.3333 for CSF to 1.4825 for metastasis as provided in supplementary S4. **(a)–(d)** Resonant frequency vs. FWHM, **(e)–(h)** S vs. Q and **(i)–(l)** DL vs. FOM for TDM_A_ and TDM_B_, respectively. All the solid lines belong to the left vertical axis, and all the dashed lines belong to the right vertical axis in **(a)-(l)**.

For Scheme-1, the resonant frequency (f_r_) slowly decreases for both TE and TM for θ_in_ = 0° to θ_in_ = 89° in Gap-1 and Gap-2. The slope of f_r_ curve for TE is slightly larger than TM in both gaps as shown in Figs. 7(a)-(d). The FWHM remains almost constant for θ_in_ = 0° in Gap-1 and increases linearly for higher θ_in_ values. In Gap-2, it remains almost constant for θ_in_ = 0° and decays exponentially towards θ_in_ = 89°. The S remains the same for all θ_in_ values in Gap-1 for TE. For TM, it remains almost the same for θ_in_ = 0° and decreases slightly for higher θ_in_ values towards the low RI region. In Gap-2, it increases both for TE and TM linearly for all θ_in_ values and towards the high RI region. The Q factor decreases for both TE and TM in Gap-1 and increases linearly in Gap-2 for high index values, as shown in Figs. 8(e)-(h). The DL increases linearly in Gap-1 for both TE and TM from the lower to the higher RI region. It decreases linearly in Gap-2 both for TE and TM. The slope of the DL curve for TE in Gap-2 is larger than TM. The FOM decreases linearly for all RI values for TE and TM in Gap-1 and increases in Gap-2, as shown in Figs. 8(i)-(l). The source data for Fig. 8 is provided in Extended Supplementary Table indicated as Scheme-1.

For Scheme-2, the resonant frequency (f_r_) slowly decreases for both TE and TM for θ_in_ = 0° to θ_in_ = 89° in Gap-1 and Gap-2. The slope of the f_r_ curve for TE is slightly larger than TM in both gaps, as shown in Figs. 7(a)-(d). The FWHM remains almost constant for θ_in_ = 0° in Gap-1 and increases linearly for higher θ_in_ values. In Gap-2, it increases exponentially for lower θ_in_ values and the high index region, both for TE and TM. The S decreases slightly from low to high RI regions for all angles in Gap-1 and Gap-2 shown in Fig. 8(e)-(h). The Q factor decreases for both TE and TM in Gap-1 and in Gap-2 for high index values linearly. The slope of the Q factor curve in Gap-1 is almost the same for TE and TM, and it becomes larger for TM than for TE in Gap-2. The DL increases linearly in Gap-1 and Gap-2 for both TE and TM from the lower to the higher RI region. The slope of the DL curve for TM in Gap-2 is larger than that of TE. The FOM decreases linearly for all RI values for TE and TM in Gap-1 and Gap-2, shown in Figs. 7(i)-(l). The source data for Fig. 7 is provided in Extended Supplementary Table indicated as Scheme-2. Note that the value for the first RI entry is omitted in Figs. 7(e)-(f) as S is undefined for no reference RI for the first sample.

Based on the results presented in the above discussion, it is evident that the proposed sensor model is highly tunable and stable. Topological protection ensures the existence and spectral stability of TDMs against structural disorder, but does not suppress material absorption or scattering losses. Our Zak-phase design guarantees TIMs and TDMs persistence under interface roughness up to σ = 3 nm, yet Q degrades from ~ 10⁹ to ~ 10⁶ (Supplementary note 7). Thus, topology governs mode existence; material losses govern linewidth. The ultra-high Q values reported represent theoretical bounds; realistic Si/SiO₂ implementations achieve Q ~ 10⁶. Each scheme has its own advantages. Polarization based comparison for both schemes is presented in Table 1.

**Table 1:** Polarization comparison summary for sensing applications.

Parameter	TE Advantage	TM Advantage	Recommendation
Q	Higher in Gap-2 at high angles	Higher in Gap-1 at normal incidence	TE for Gap-2 sensing
S	More stable across angles	Higher absolute values	TM for maximum S
FOM	Better at high RI	Better at low RI	TE for high-RI samples
Practical use	Robust against alignment	Efficient signal	TE for field deployment

For example, one can achieve high Q by Scheme-1, and obtain high S by Scheme-2. The Table 1 summarizes: (i) Q: TE performs better in Gap-2 at high incident angles, while TM is superior in Gap-1 at normal incidence; (ii) S: TE provides more stable response across angles, whereas TM achieves higher absolute S values; (iii) FOM: TE is preferred for high RI samples, TM for low RI; and (iv) Practical deployment: TE is recommended for field applications due to alignment robustness, while TM offers higher signal strength. These guidelines enable informed polarization selection based on specific application requirements.

The detailed sensing parameter values can be seen in the supplementary information Extended Supplementary Tables Scheme-1 and Scheme-2. A brief comparison with the recent works for 1D TPC is also presented in Table 2. The best sensing parameter values obtained for Scheme-1as Q, DL and FOM for θ_in_ = 89° in Gap-2 for TE mode and for Scheme-2 the best S values obtained for Gap-2 for TE mode at θ_in_ = 89°.

**Table 2:** A comparison with recent work for 1D TPC biosensors reported in literature.

Ref.	Wavelength (nm)	S (nm/RIU)	Q	DL (RIU)	FOM (RIU^− 1^)
^17^	1700	603.753	2.33 ⤫10^4^	1.22⤫10^− 4^	8147.814
^28^	1521	852.14	4019.23	--	1277.13
^29^	1900	1053.200	55284.800	--	29897.143
^19^	--	950	5870	--	161
This work	1548.6	397.7 THz/RIU or 3179.56 nm/RIU	7.24⤫10^9^	7.6⤫10^− 11^	1.32 ⤫10^9^

The experimental validation for the theoretical predictions is outlined in the Supplementary Note 7 that includes a comprehensive setup for fabricating and measuring the performance of the proposed device with a focus on their sensitivity and robustness.

## Conclusion

We present a thorough investigation of all-dielectric 1D TPCs comprising Si and SiO_2,_ focusing on their tunability, robustness, and stability. Leveraging on the PGC strategy we achieve robust and strongly confined TIMs with superior sensitivity. The TIMs and TDMs remain within the topological operation region when the incident angle is varied from 0° to 89° both for TE and TM modes. By defect engineering, we presented two schemes to achieve TDMs for high tunability and high sensitivity for RI sensing. These two schemes demonstrate the superior performance defined by the parameters S, Q, DL, and FOM.

These performance metrics represent two distinct regimes: (i) theoretical upper bounds derived under ideal lossless conditions to establish the fundamental potential of the PGC design, and (ii) realistic projections incorporating material absorption and fabrication imperfections. Under ideal conditions, our transfer matrix calculations predict the high Q of 7.24⤫10^9^ and ultra-low DL of 7.6⤫10^–11^ RIU, with S of 397.7 THz/RIU or 3179.56 nm/RIU and a high FOM of 1.32 ⤫10^9^ RIU^−1^. However, when realistic material losses are included through frequency-dependent complex RIs for Si and SiO₂ (Supplementary Note 3), and interface roughness scattering is modeled using the Nevot-Croce formalism (Supplementary Note 7, Fig. S9), the performance metrics reduce to Q ~ 10⁶ and DL ~ 10⁻⁷ RIU for surface roughness σ ≈ 0–3 nm. These values align with experimentally demonstrated performance in comparable Si/SiO₂ distributed Bragg reflector cavities^29,30^.

We emphasize that topological protection guarantees the existence and spectral stability of interface modes against structural disorder, but does not eliminate intrinsic material absorption or scattering losses, a distinction crucial for practical device implementation. Additional reductions in Q-factor from radiation leakage in finite structures and fabrication disorder would be expected in experimental realizations, though the topological robustness ensures mode persistence even under these perturbations. The sensitivity (S ~ 3000 nm/RIU) remains attractive for biosensing applications even with realistically achievable Q-factors of ~ 10⁶.

## Supplementary Information

Below is the link to the electronic supplementary material.

Supplementary Material

## Data Availability

All data generated or analyzed during this study are included in the paper, the Extended Data sets indicated as Scehem-1 and Scheme-2 and the Supplementary Information. All codes related to this study are available on publicly accessible repository https://github.com/RSKashmiri/sr-topo-source-code/tree/main. Additional queries can be directed to the corresponding authors.
