# Supplementary material for: Ultrasensitive topological interface modes for refractive index sensing via all-dielectric one-dimensional photonic crystals
Source: Sci Rep. 2026 Apr 9;16:16979. doi: 10.1038/s41598-026-47618-z (PMC13230802; doi:10.1038/s41598-026-47618-z)
Supplement: Supplementary file 1 — Supplementary Material [file 41598_2026_47618_MOESM1_ESM.docx]

# Supplementary information for

**Ultrasensitive topological interface modes for refractive index sensing via all-dielectric one-dimensional photonic crystals**

**Shakeel Ahmed^1^, Muhammad Zeeshan Riaz^2^, Saad Anwar^1^, Maryam Jamil^1^, Juncong Luo^1^, Mi Lin^1^*, and Zhengbiao Ouyang^1^***

*^1^Key Laboratory of Optoelectronic Devices and Systems of the Ministry of Education and Guangdong Province, College of Physics and Optoelectronic Engineering, Shenzhen University, Shenzhen 518060, China*

*^2^Intelligent Optical Measurement Research Institute, Shenzhen University, Shenzhen 518061, China*

** Corresponding Authors:* [*linfengas111@szu.edu.cn, zbouyang@szu.edu.cn*](mailto:linfengas111@szu.edu.cn,%20zbouyang@szu.edu.cn)

## Contents

## Supplementary Figures S1-S9

## Supplementary Tables S1-S2

**Supplementary Note 1: The band structure and transmission spectra of centrosymmetric TPCs**…2

**Supplementary Note 2: The reflection phases and band topology of centrosymmetric TPCs**………..2

**Supplementary Note 3: The material properties in considered frequency range**…………………...4-5

**Supplementary Note 4: Thickness optimization** **and transmission for Scheme-1 and Scheme-2**…..............................................................................................................................................................5-6

**Supplementary Note 5: E and H field profiles of TPCs** …………....................................................6-7

**Supplementary Note 6: Brain tumor samples and their categories** …………....................................7

**Supplementary Note 7: Experimental proposal** ………….................................................................7-9

**References**………………………………………………………………………………………………….9

**Supplementary Note 1: The band structure, transmission spectra of CPCs**

The band structures of PC_1_ (P) and PC_2_ (Q) are calculated using equ. (1) of the main text and presented in Figs. S1(b) and (c) respectively, and the corresponding transmission spectra are presented in Fig. S1(a). Figure S1(a) explains the existence of alternate topological interface modes (TIMs) in Gap-1 and Gap-2 indicated as TIM_A_ and TIM_B_ in the main text appear to be positioned below and above the base frequency $f_{0}$ i.e., f _TIMA_<$f_{0}$ in Gap-1 and f _TIMB_>$f_{0}$ in Gap-2. The TIMs behavior of such a centrosymmetric structure can be explained using the equ. (9) of the main text.


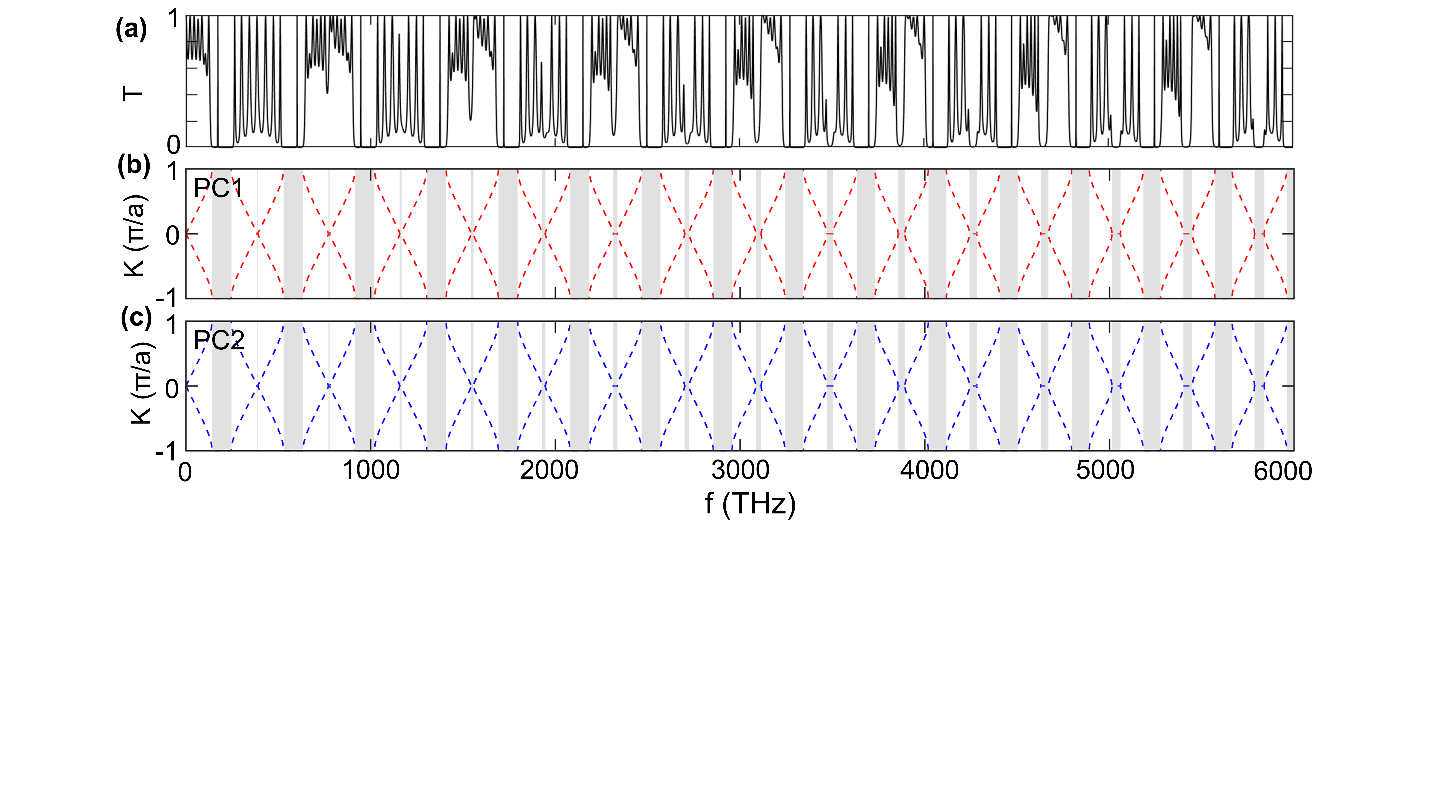


**Fig. S1. Dispersion and transmission profiles of P and Q.** (a) The transmission spectrum for P and Q from 0 to 6000 THz. (b) and (c) are the band structures of P and Q, respectively. The odd and even gaps tend to decrease and increase linearly across the frequency spectrum. Note that the higher order TIMs are in quasi-periodic manner according the Equ. (9) of the main text.

**Supplementary Note 2: The reflection phases and band topology of centrosymmetric TPCs**

In our study we considered the low-loss, isotropic and non-magnetic dielectric materials for PCs P and Q which are experimentally well tested in the THz band. When a plane wave of frequency f (with angular frequency ω=2πf) is incident at the an angle θ, each semi-infinite PC acts like a mirror inside the stop band with unit-magnitude of reflection coefficient;

$r_{i}\left( f, \theta\right)=e^{{iФ}_{i}(f,\theta)}$, |$r_{i}|=1, i=1,2$, (Equation.S1)

This can be recast in the form of surface impedance as;

$Z_{i}\left( f, \theta\right)=Z_{o}\frac{1+r_{i}}{1-r_{i})}$ $= iZ_{o}cot\left( \frac{Ф_{i}}{2} \right)$, (Equation.S2)

where $Z_{o}$ is the impedance of the incidence medium. A localized bounded mode exists at the interface if the boundary conditions at z = 0 can be satisfied without external excitations that is,

$Z_{1}\left( f, \theta\right)= Z_{2}\left( f, \theta\right)=0.$ (Equation.S3)

Substituting Eq.S2 in Eq.S1 reduces it to

$cot\left( \frac{Ф_{1}}{2} \right)$+ $cot\left( \frac{Ф_{2}}{2} \right)=0,$ (Equation.S4)

which is equivalent to,

$sin\left( \frac{{Ф_{1}+Ф}_{2}}{2} \right)=0 \LeftrightarrowФ_{1}\left( f, \theta\right)+ Ф_{2}\left( f, \theta\right)=2m\pi.$ (Equation.S5)

Equation S5 satisfies the condition of the two reflectivities $r_{1}r_{2}=1$ that is their phase sum is an integer multiple of 2π.


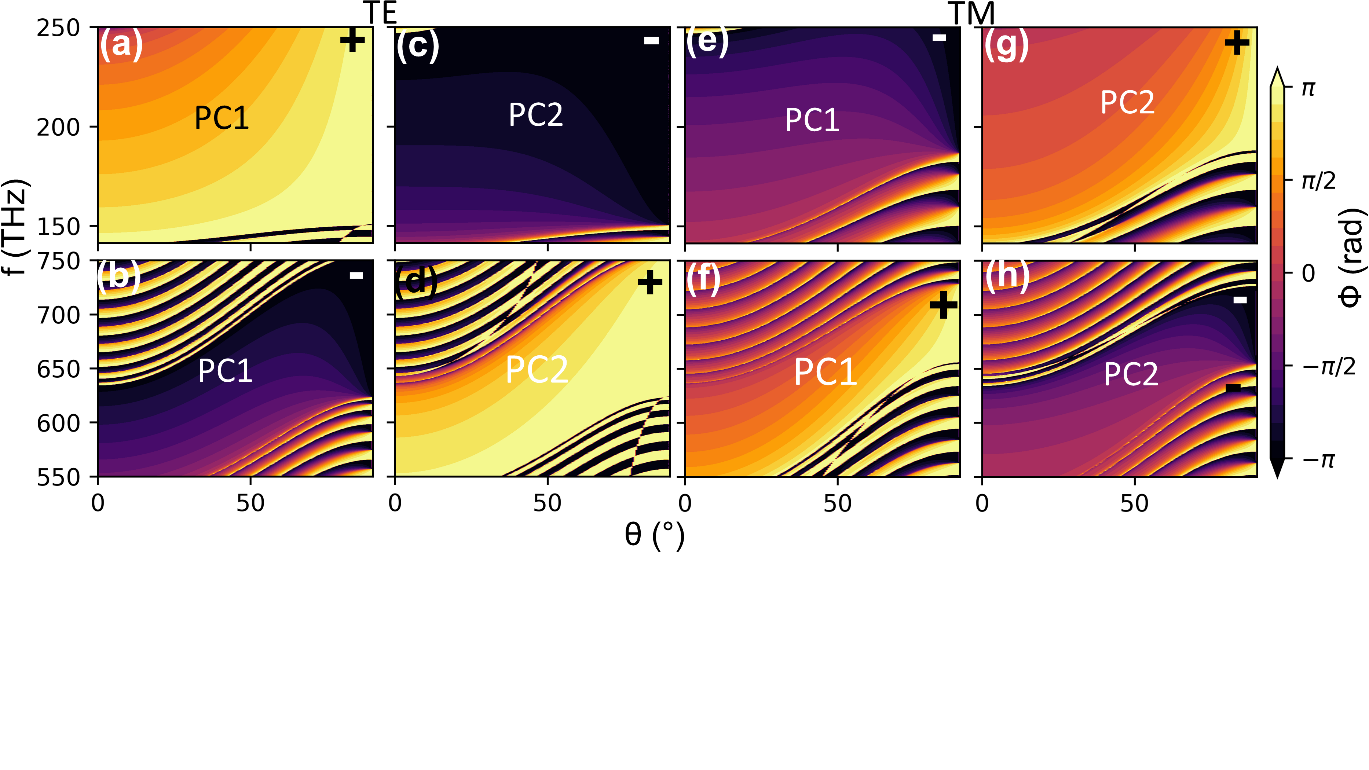


**Fig. S2. The reflection phase map of individual PCs.** The reflection phase map of isolated PCs P and Q in (a)-(d) for TE and (e)-(h) for TM. The first and second rows represent the in Gap-1 and Gap-2 phases, respectively.

Now let us define,

$F\left( f, \theta\right)\equivФ_{1}\left( f, \theta\right)+ Ф_{2}-2m\pi.$ (Equation.S6)

The TIM dispersion $f_{TIM}\left( \theta\right)$ is the implicit solution of F = 0. Differentiating along the solution curve and applying the chain rule gives,

$0=\frac{dF}{d\theta}=\left[ \left( \partial_{\theta}Ф_{1}+ \partial_{\theta}Ф_{2} \right)+\left( \partial_{f}Ф_{1}+ \partial_{f}Ф_{2} \right) \right]\frac{dF_{TIM}}{d\theta}$ . (Equation.S7)

Solving for slope gives,

$\frac{{df}_{TIM}}{d\theta}=-\frac{\partial_{\theta}Ф_{1}+\partial_{\theta}Ф_{2}}{\partial_{f}Ф_{1}+\partial_{f}Ф_{2}} .$ (Equation.S8)

The denominator in Eq. S8 directly links the reflection group delays for PCs L and R. Since$\partial_{f}Ф_{i}=2\pi\partial_{\omega}Ф_{i}=2\pi\tau_{i}$, one gets

$\partial_{f}Ф_{1}+\partial_{f}Ф_{2}=2\pi($ $\tau_{1}+\tau_{2}$). (Equation.S9)

In the Eq. S9 the term $\tau_{i}$ refers to the Wigner-Smith[1,2] delay from mirror *i* (P-Q system), that is, the time that a mirror *i* retains the incoming wave before it re-radiates the energy back into the medium. Increasing the total delay $\tau_{1}+\tau_{2}$ can enhance the stop-band strength (for example, by index contrast and optimizing the optical filling ratio), choosing the frequency bounded tightly within the stopband to amplify the retro-reflected interference. When the cumulative delay is larger, the fractional change rate for the wave-vector in the transfer matrix drops, leading to a narrower output beam defined in angular space. Therefore, the choice of material plays a crucial role in not only achieving a stop-band in a particular frequency window but also the angle dependence consequently controlling the tunability and sensitivity properties of device. In contrast, the nominator, that is the cumulative angular phase, that enters the phase-matching condition via Snell's law, along with the layered equivalent Fresnel parameters, so, ${Ф_{i}=Ф}_{i}\left( f, \theta\right)$ varies with$\theta$. When the angular phase slopes of the two facing mirrors are tailored to cancel across the common gap, the numerator of in Equ. S8 minimizes to ensure the consistent existence of TIMs. The reflection phase profiles for isolated PCs P and Q are presented in Fig. S2. Table S1 presents the Zak phase and reflection phase for considered range of first four gaps.

**Table S1: The table showing the reflection phase *Ф_n_*, and Zak phase** $\boldsymbol{\theta}_{\boldsymbol{m}}^{\boldsymbol{zak}}$ **in the first 4 PBGs.** [3]

|  | PC1:P | | PC2:Q | | TIM existence |
| --- | --- | --- | --- | --- | --- |
| PBG  (Band) | θ_Zak_ | Sgn(***Ф_n_***) | θ_Zak_ | Sgn(***Ф_n_***) |  |
| 4  (3) | 0 | + | π | + | No |
| 3  (2) | π | - | 0 | + | Yes |
| 2  (1) | 0 | - | π | - | No |
| 1  (0) | π | + | 0 | - | Yes |

**Supplementary Note 3: The material properties in considered frequency range**

In our simulations we incorporated the material parameters including complex refractive indices for Si and SiO_2_ derived from established experimental data [4–9], and systematically model fabrication imperfections (Scheme-II) such as layer thickness variations and interface adjacent layer deletion (Scheme-I). In the calculated n and k plots for Si and in Fig. S3, the behavior of the materials over the frequency ranges of Gap-1 and Gap-2 reveals distinct trends. For Si, the n remains relatively constant in the lower frequency range and increases with frequency in the higher range reflecting the expected behavior of a semiconductor with dispersive properties due to inter-band transition of Si, particularly at the visible and near-infrared regions.


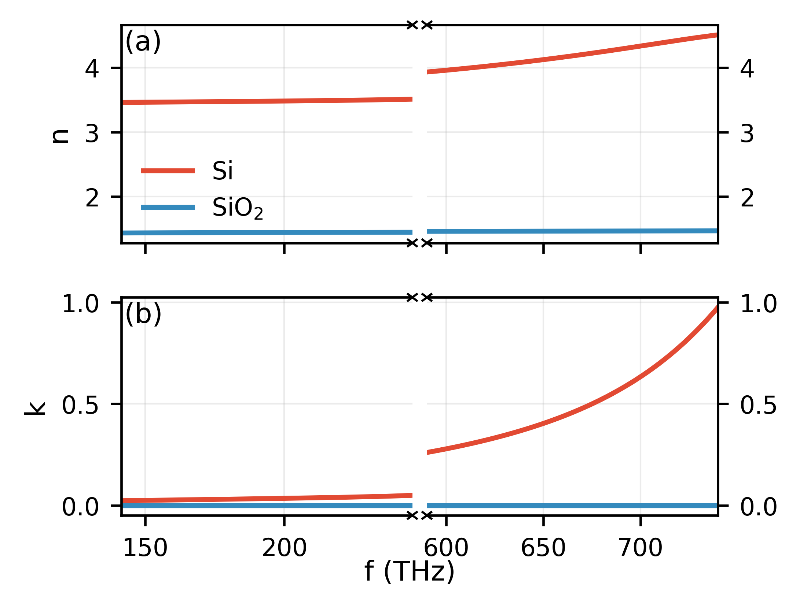


**Fig. S3.** (a) Frequency dependent refractive indices n of Si and SiO_2_ and (b) extinction coefficient k in the considered frequency ranges.

On the other hand, for, the n is relatively stable across both ranges, consistent with experimental observations where SiO_2_ is known to have a nearly constant n (≈ 1.45) in the visible to near-infrared range, with an extremely low extinction coefficient (k ≈ 10^-5^). This behavior aligns with the characterization of SiO_2_ as a transparent material with minimal absorption, which is why k remains close to zero throughout the frequency spectrum in Fig. S3. These trends are consistent with reported values from refractive index databases [8,9] and highlight the optical contrasts between the two materials, which are essential for applications in photonic crystal designs and optical wave guiding systems. Therefore one can conclude that the results presented in our study are close enough to realistic ones obtained in the experiments.

### Supplementary Note 4. Thickness optimization and transmission for Scheme-1 and Scheme-2

To find the maximum transmittance regions across the frequency spectrum the thickness is optimized from 1- 1000 nm. Multiple spans of optimized thickness for high transmittance are found in both gaps for both the Scheme-1 and Scheme-2 for normal incidence as shown in Fig. S4.


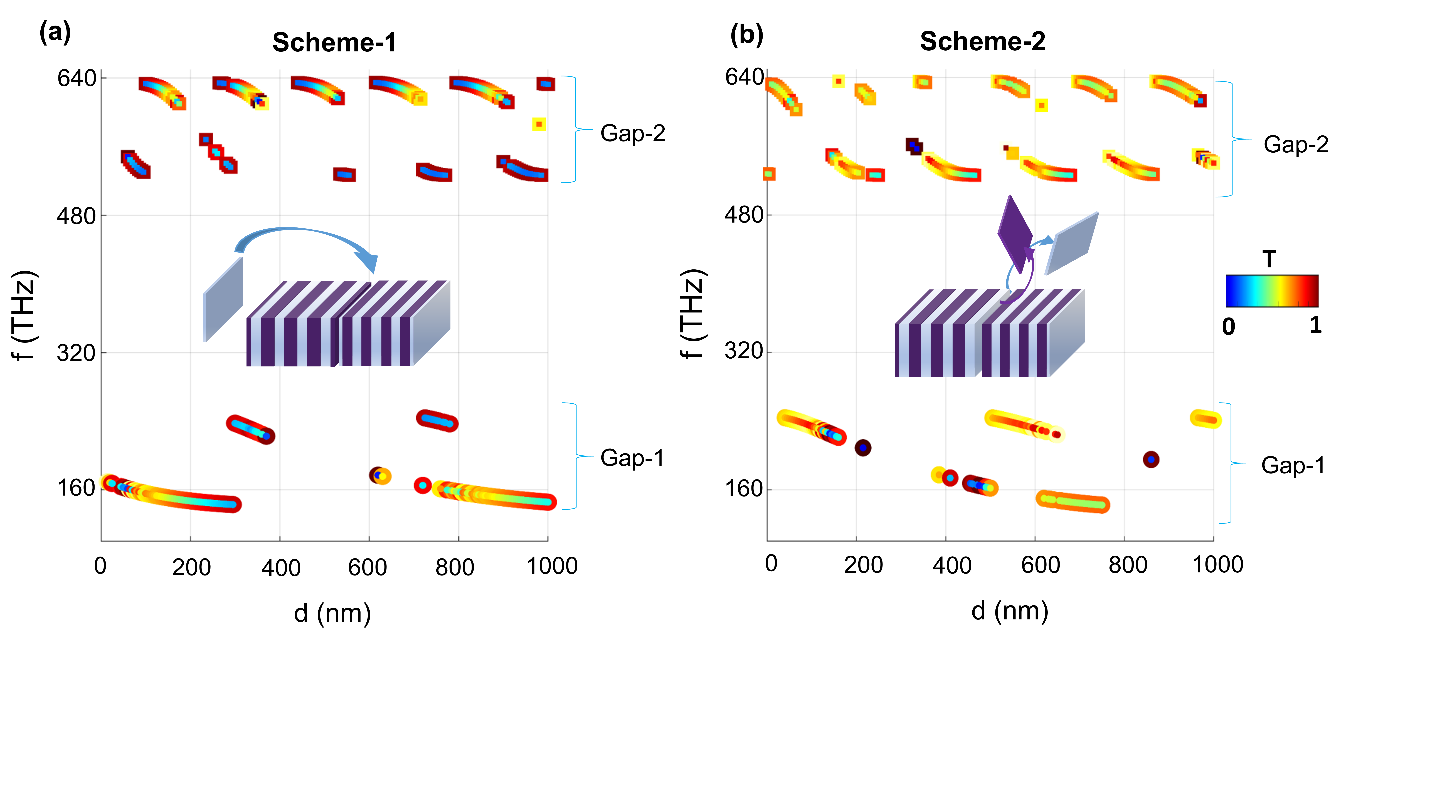


**Fig. S4. Thickness optimization.** (a) represents the optimized thickness for Scheme-1 and (b) for Scheme-2 respectively, for normal incidence.

For instance, in the first span in the range 180 to 195 nm the transmittance becomes greater than 90% with peak value at 190 nm (95%) for Scheme-1. Similarly for Scheme-2, the maximum transmittance comes in the range 370 nm to 390 nm with the peak value at 380 nm. The spans repeat after regular intervals in both gaps. Note that the transmittance will be lower than the maximum value on either side of these spans. The defect thickness for refractive index sensing was taken to be 190 nm for Scheme-1 and 380 nm for Scheme-2 in the main text. The corresponding transmittance profiles for Scheme-1 and Scheme-2 are provided in Fig. S5


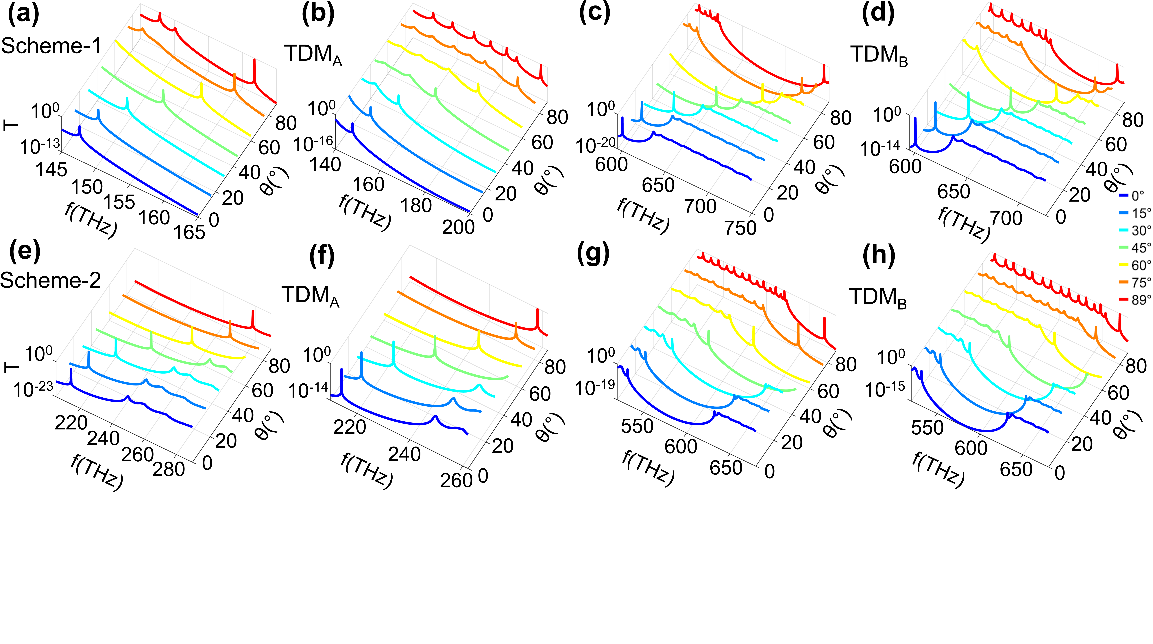


**Fig. S5. The profiles for** **Scheme-1 and Scheme-2.** (a)-(d) The T profiles of TDM_A_ and TDM_B_ for TE and TM modes for Scheme-1, respectively. (e)-(h) The T profiles of TDM_A_ and TDM_B_ for TE and TM modes for Scheme-2, respectively.

For Scheme-1 the TDMs for both gaps shift but remain within the topological operating region across the T spectrum, as observed in Supplementary Fig. S6(a)–(h). It is seen that for TDM_A_, the TE and TM modes behave differently. For TE, the T decreases slightly towards higher θ_in_ values, whereas it increases for TM. For TDM_B_, the T behaves similar to that in Gap-1 for TE and TM from 0° to 89°. However, the overall T is slightly lower in Gap-2 than in Gap-1. For Scheme-2 the TE modes in Gap-1 behave similarly to Scheme-1 but with less T. For TM modes, the T behaves similarly to TE across the θ_in_ scan but with lower values than TE. In Gap-2, the T decreases slightly for higher θ_in_ for TE and increases for TM. The overall T in Gap-2 is more than in Gap-1 for both TE and TM polarizations.

### Supplementary Note 5. E and H fields for Scheme-1 and Scheme-2

### Both E and H fields are observed to be confined around the interface. Since in both PCs, P and Q, the TIMs frequency is well inside the PBG so the Bloch wave is evanescent on either sides of the interface.

Since the reflection phases form P and Q satisfy Eq. S5, while local impedances have positive imaginary parts therefore energy decays away from the interface. The confinement length scales at $l^{-1}\approx\kappa_{1}+\kappa_{2},$ with $\kappa$being the Bloch wave number. Hence stronger gaps yield more confinement. The polarization dependence follows $p_{TE}=\sqrt{\frac{\varepsilon}{\mu}}cos\theta_{l}$ and $p_{TM}=\sqrt{\frac{\mu}{\varepsilon}}cos\theta_{l}$ that means changing θ_in_ alters the normal component of the wave vector and hence the impedance contrast across the interface. Therefore the TIMs frequency blue shifts with angle while both E and H fields drop with the penetration depth increases. The TIMs act as tunneling channel that concentrates energy at the interface while leaking symmetrically to the free space. Figure S6 and S7 demonstrates the E and H fields for Scheme-1 and Scheme-2 respectively. A shift in excitation frequencies justifies the consistent localization of the mode field near the interface. The H field shows more confinement and higher responsiveness than its counter-part as can be seen in Fig. S6 and Fig. S7 for both schemes. The E and H field confinement in Gap-2 in both schemes justifies the ultra-high Q reported in the main text.


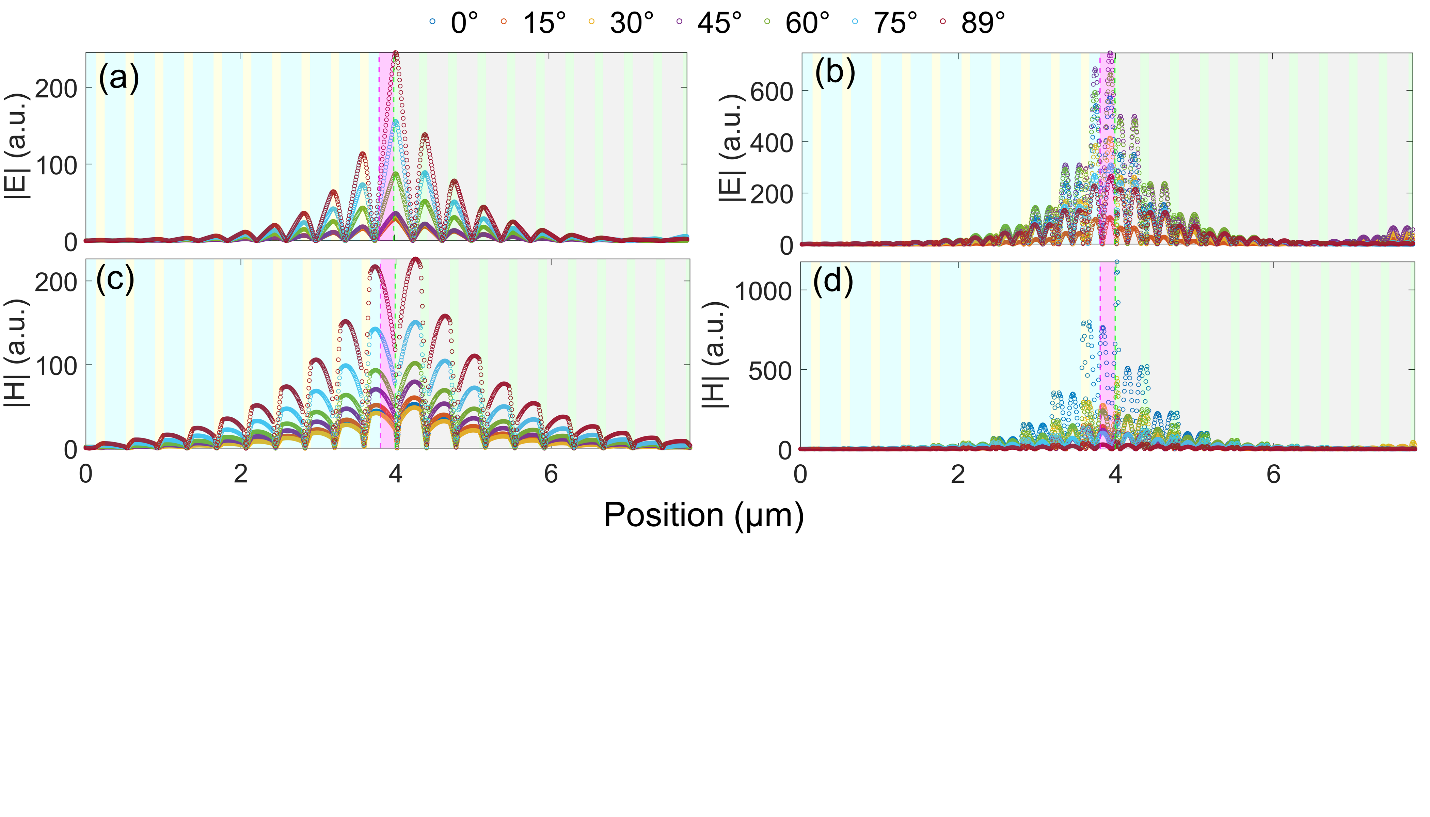
 **Fig. S6. E and H field profiles for Scheme-1.** The E and H field profiles of TDM_A_ in (a), (c) and TDM_B_ in (b),(c) respectively for Scheme-1.

###
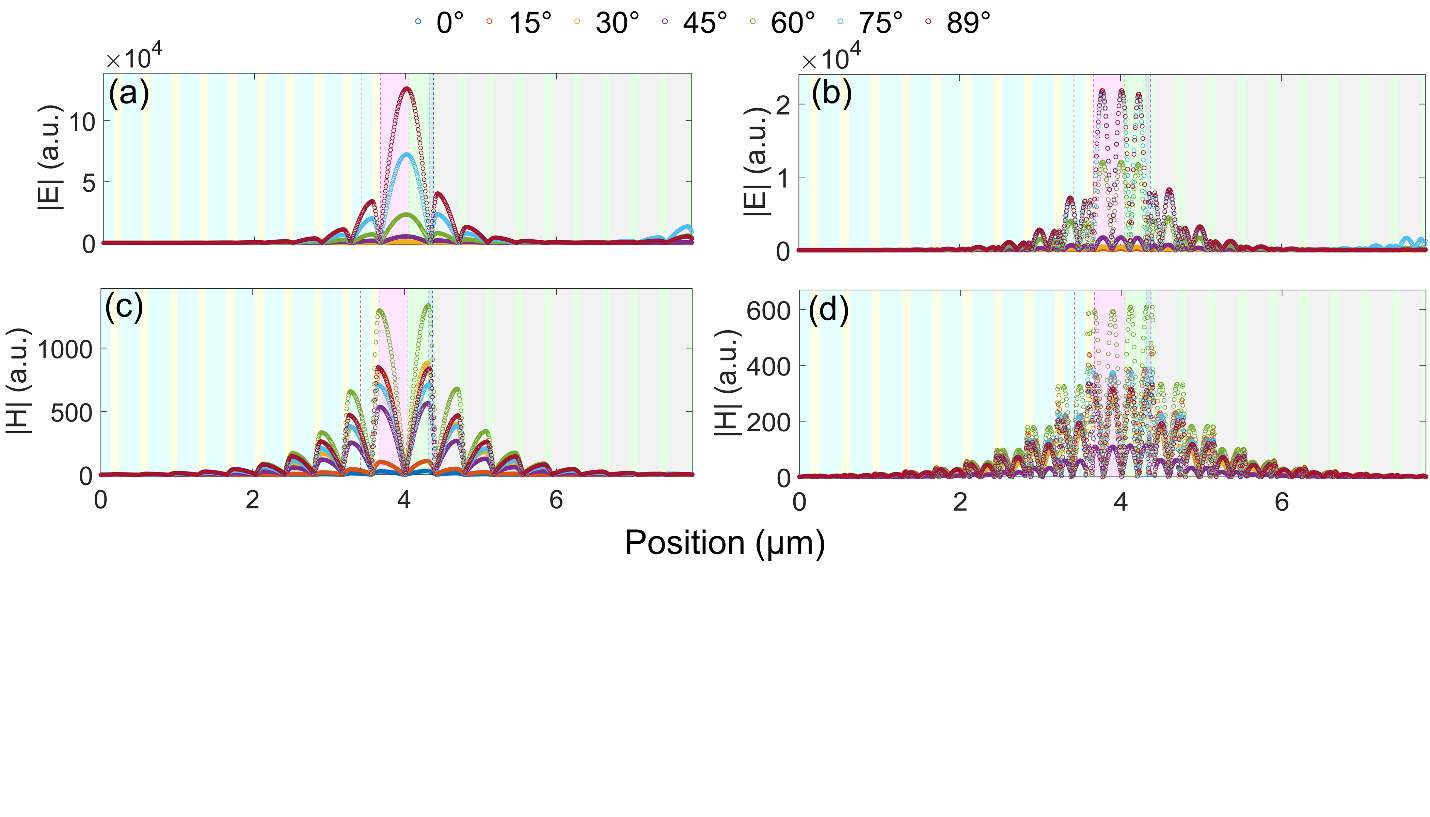


**Fig. S7. E and H field profiles for Scheme-2.** The E and H field profiles of TDM_A_ in (a), (c) and TDM_B_ in (b),(c) respectively for Scheme-2.

### Supplementary Note 6. Brain tumor samples and their categories

The brain tumor samples information provided here is adopted from ref. [10]. These samples have been used as refractive index variable in both the schemes discussed in the main text. The details of RIs for samples and their categories are provided in the Table S2.

**Table S2. The refractive index of the brain tumor samples used in refractive index sensing in the main text.** [10]

| Brain samples | | | | Refractive index |
| --- | --- | --- | --- | --- |
| Normal tissues | | | CSF | 1.3333 |
|  |  |  | Gray matter | 1.3951 |
|  |  |  | White matter | 1.4121 |
| Abnormal tissues | Injured tissues (lesions) | | Wall of solid brain | 1.3412 |
|  |  |  | Multi sclerosis | 1.3425 |
|  |  |  | Oligodendroglioma | 1.3531 |
|  | Tumors and cancers | Benign | Low grade glioma | 1.4320 |
|  |  | Cancers | Medulloblastoma | 1.4412 |
|  |  |  | Glioblastoma | 1.4470 |
|  |  |  | Lymphoma | 1.4591 |
|  |  |  | Metastasis | 1.4833 |

### Supplementary Note 7. Experimental Proposal

The experimental verification of the proposed 1D-TPC based on the PGC model can be performed using a terahertz time-domain spectroscopy (THz-TDS) setup, as schematically shown in Fig. S8. A THz emitter (0-800 THz) can be used as the source to collimate the beam and spatially filter by a combination of a parabolic mirror, lens, and iris diaphragm. A wire-grid polarizer defines the TE or TM polarization before the beam impinges on the fabricated 1D PGC sample mounted on a rotation stage for precise control of the incident angle (0°-89°). The transmitted signal is refocused by another parabolic mirror and collected by a pyroelectric detector or THz receiver, with spectral data acquired using a fiber-coupled spectrometer. The sample consists of alternating Si and SiO_2_ layers arranged in the CPC configuration can be fabricated via hot-press lamination at, followed by SEM and interferometric verification of uniformity (±3%). Transmission spectra can be recorded for both TE and TM modes from 0° to 89° and normalized against a reference path. The results are expected to show distinct TIMs with predicted angular dependence and polarization sensitivity. For the RI sensing the interface can be loaded with samples provided in the Table S2.


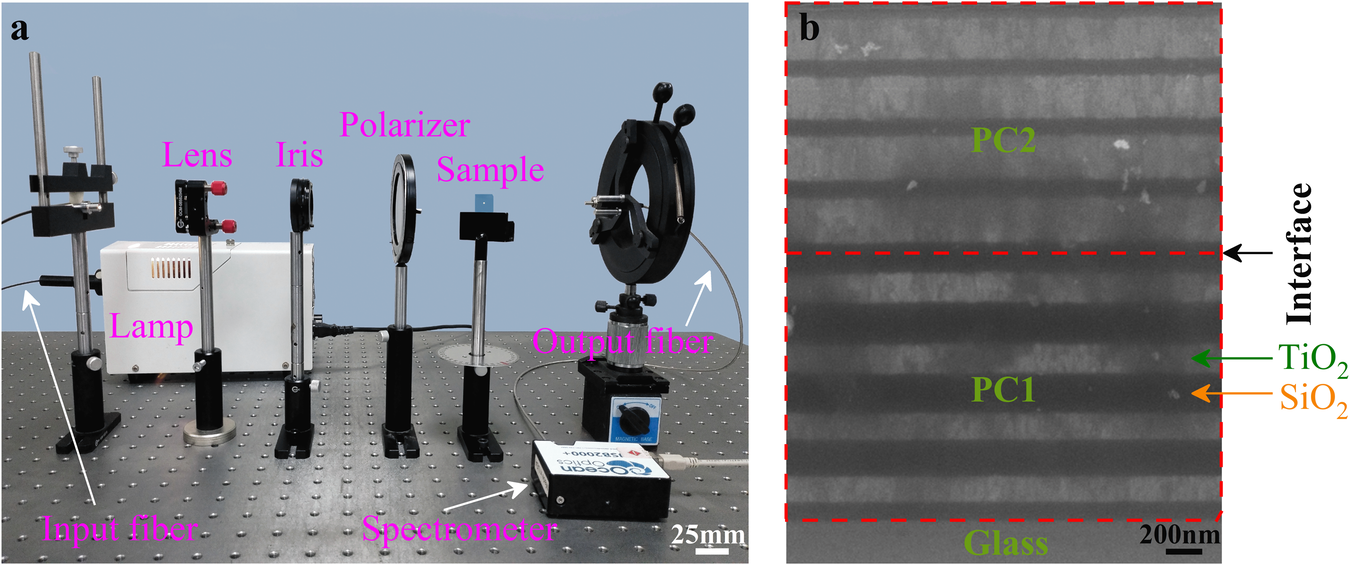


**Fig. S8.** **Experimental setup.** The proposed experimental setup to carryout transmission spectroscopy and angle dependence of TIMs.


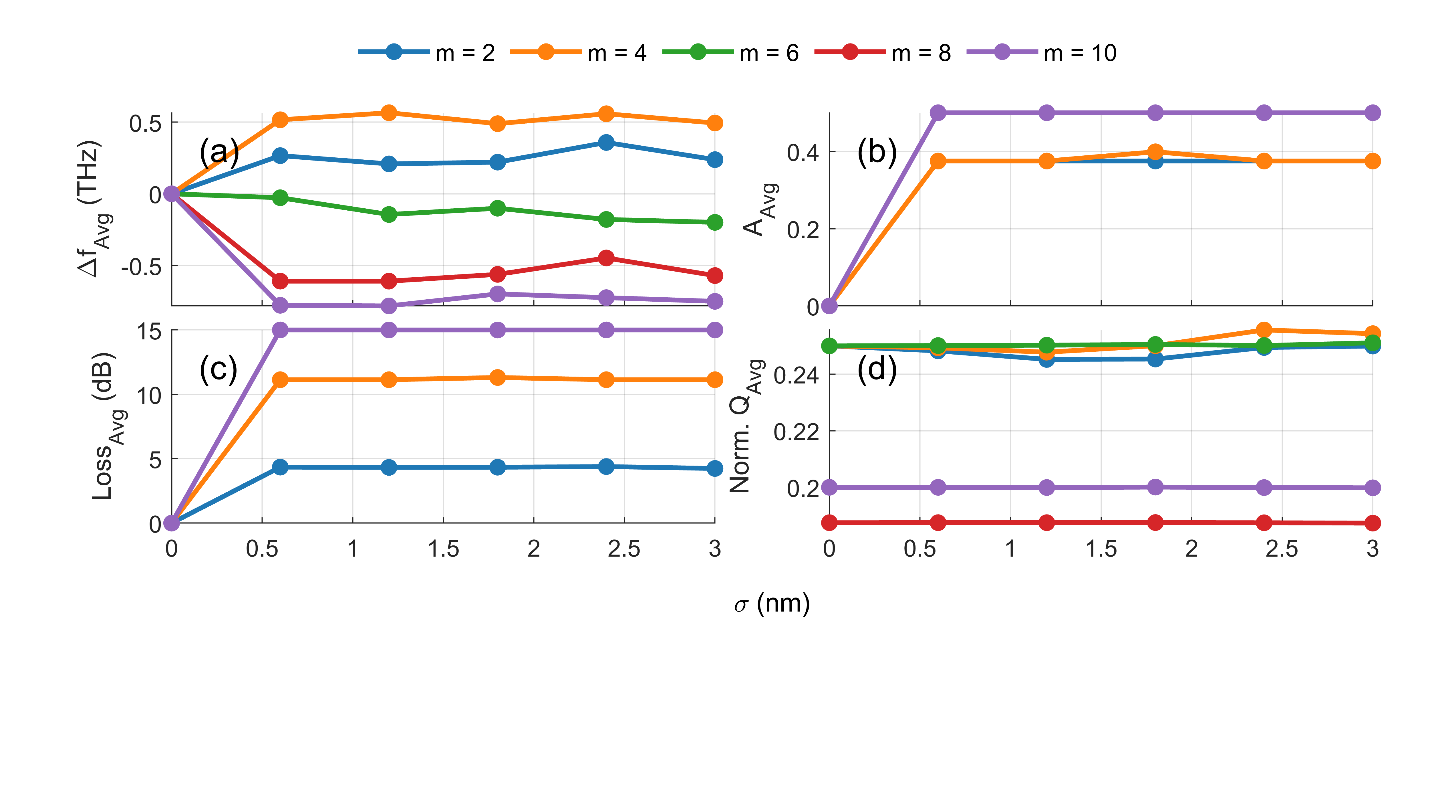


**Fig. S9.** **Performance metrics under imperfect surface conditions.** (a) Frequency shift (b) average absorption (c) Average optical loss (d) normalized Q, for 2-10 periods under layer roughness up to 3 nm.

The performance metrics given in the main text are theoretical limits of our proposed model, representing upper bounds derived under ideal conditions to demonstrate the fundamental potential of the Si/SiO_2_ topological Bragg stack. To quantify realistic degradation, we modeled dominant losses as shown in Fig. S9 via interface-roughness scattering using the Nevot–Croce (NC) model, applied layer-by-layer with surface roughness σ from 0 - 3 nm [11]. As illustrated in Fig. S9, imperfections elevate optical loss to ~15 dB (Fig. S9 (c)), reduce Q from ~10^9^ to ~10^6^ (Fig. S9 (d)), increase absorption (Fig. S9 (b)), and mildly impair TIMs (Fig. S9 (a)). While TIMs remain robust, topology does not negate absorption or scattering, aligning with practical expectations.

##### References

1. F. T. Smith, "Lifetime matrix in collision theory," Phys Rev (118), 349 (1960).

2. E. P. Wigner, "Lower limit for the energy derivative of the scattering phase shift," Phys Rev **98**, 145 (1955).

3. W. Gao, M. Xiao, B. Chen, et al., "Controlling interface states in 1D photonic crystals by tuning bulk geometric phases," Opt Lett **​42**, 1500–1503 (2017).

4. Liu, Y., Li, H., Tong, H., et al., "Rational design and construction of [Si/SiO2] N one-dimensional photonic crystal for low infrared emissivity and visible light camouflage," Opt. Lasers Eng. **167**, 107641 (2023).

5. Palik, Edward D., ed., *Handbook of Optical Constants of Solids*, Academic press, (1998), **3**.

6. Kim, C., Ahn, J. S., Ji, T., et al., "Terahertz transmission properties of silicon wafers using continuous-wave terahertz spectroscopy. Measurement Science and Technology," **28(4)**, 045201 (2017).

7. Kitamura, R., Pilon, L., & Jonasz, M., "Optical constants of silica glass from extreme ultraviolet to far infrared at near room temperature. Applied optics," **46(33)**, 8118–8133 (2007).

8. https://refractiveindex.info/

9. Polyanskiy, Mikhail N., "Refractiveindex. info database of optical constants," Sci. Data **11.1**, 94 (2024).

10. N. A. Mohammed, M. Elhusseiny, Z. Y. Mohamed, et al., “Brain tumors biomedical sensor with high-quality factor and ultra-compact size based on nanocavity 2D photonic crystal,” Alex. Eng. J. 64, 527–540 (2023).

11. Nevot, 11_L, and P. Croce., "Caractérisation des surfaces par réflexion rasante de rayons X. Application à l’étude du polissage de quelques verres silicates," Rev. Phys. Appliquée **15.3**, 761–779 (1980).
